# Biparatopic antibody BA7208/7125 effectively neutralizes SARS-CoV-2 variants including Omicron BA.1-BA.5

**DOI:** 10.1038/s41421-022-00509-9

**Published:** 2023-01-07

**Authors:** Yanqun Wang, An Yan, Deyong Song, Chuangchuang Dong, Muding Rao, Yuanzhu Gao, Ruxi Qi, Xiaomin Ma, Qiaoping Wang, Hongguang Xu, Hong Liu, Jing Han, Maoqin Duan, Shuo Liu, Xiaoping Yu, Mengqi Zong, Jianxia Feng, Jie Jiao, Huimin Zhang, Min Li, Beibei Yu, Yanxia Wang, Fanhao Meng, Xiaodan Ni, Ying Li, Zhenduo Shen, Baiping Sun, Xin Shao, Haifeng Zhao, Yanyan Zhao, Rui Li, Yanan Zhang, Guangying Du, Jun Lu, Chunna You, Hua Jiang, Lu Zhang, Lan Wang, Changlin Dou, Zheng Liu, Jincun Zhao

**Affiliations:** 1 State Key Laboratory of Respiratory Disease, National Clinical Research Center for Respiratory Disease, Guangzhou Institute of Respiratory Health, the First Affiliated Hospital of Guangzhou Medical University, Guangzhou, Guangdong China; 2 GMU-GIBH Joint School of Life Sciences, Guangzhou Medical University, Guangzhou, Guangdong China; 3 Cryo-electron Microscopy Center, Southern University of Science and Technology, Shenzhen, Guangdong China; 4 Antibody Research and Development Center, Shandong Boan Biotechnology Co., Ltd., Yantai, Shandong, China; 5 Division of Monoclonal Antibodies, Institute for Biological Product Control, National Institutes for Food and Drug Control (NIFDC), Beijing, China; 6 Shuimu BioSciences Ltd, Beijing, China; 7 State Key Laboratory of Long-acting and Targeting Drug Delivery System, Shandong Luye Pharmaceutical Co. Ltd, Yantai, Shandong, China; 8 Health and Quarantine Laboratory, Guangzhou Customs District Technology Centre, Guangzhou, Guangdong, China; 9 Guangzhou Laboratory, Bio-Island, Guangzhou, Guangdong, China; 10 Institute of Infectious disease, Guangzhou Eighth People’s Hospital of Guangzhou Medical University, Guangzhou, Guangdong China; 11 Shanghai Institute for Advanced Immunochemical Studies, School of Life Science and Technology, ShanghaiTech University, Shanghai, China; 12 Institute for Hepatology, National Clinical Research Center for Infectious Disease, Shenzhen Third People’s Hospital; The Second Affiliated Hospital, School of Medicine, Southern University of Science and Technology, Shenzhen, Guangdong China

**Keywords:** Immunology, Molecular biology

## Abstract

SARS-CoV-2 Omicron subvariants have demonstrated extensive evasion from monoclonal antibodies (mAbs) developed for clinical use, which raises an urgent need to develop new broad-spectrum mAbs. Here, we report the isolation and analysis of two anti-RBD neutralizing antibodies BA7208 and BA7125 from mice engineered to produce human antibodies. While BA7125 showed broadly neutralizing activity against all variants except the Omicron sublineages, BA7208 was potently neutralizing against all tested SARS-CoV-2 variants (including Omicron BA.1–BA.5) except Mu. By combining BA7208 and BA7125 through the knobs-into-holes technology, we generated a biparatopic antibody BA7208/7125 that was able to neutralize all tested circulating SARS-CoV-2 variants. Cryo-electron microscopy structure of these broad-spectrum antibodies in complex with trimeric Delta and Omicron spike indicated that the contact residues are highly conserved and had minimal interactions with mutational residues in RBD of current variants. In addition, we showed that administration of BA7208/7125 via the intraperitoneal, intranasal, or aerosol inhalation route showed potent therapeutic efficacy against Omicron BA.1 and BA.2 in hACE2-transgenic and wild-type mice and, separately, effective prophylaxis. BA7208/7125 thus has the potential to be an effective candidate as an intervention against COVID-19.

## Introduction

SARS-CoV-2, the causative agent of coronavirus disease 2019 (COVID-19), has caused a devastating global pandemic, resulting in more than 630 million reported cases and over 6.5 million deaths as of November 2022 (https://covid19.who.int/). Neutralizing monoclonal antibodies (nmAbs) are an important class of antiviral intervention against SARS-CoV-2. A number of nmAbs, either used as an monotherapy or a cocktail, have been granted Emergency Use Authorization (EUA) or approval for therapeutic or preventive use in the last 12 months, including bamlanivimab (LY-CoV555)^1^ & etesevimab (CB6)^2^, imdevimab (REGN10987) & casirivimab (REGN10933)^3^, tixagevimab (COV-2-2196) & cilgavimab (COV-2-2130)^4^, sotrovimab (VIR-7381)^5^, and amubarvimab (Brii-196) & romlusevimab (Brii-198)^6^. However, new variants of SARS-CoV-2 continue to emerge, including B.1.1.7 (Alpha)^7^, B.1.351 (Beta)^8^, P.1 (Gamma)^9^, B.1.621 (Mu)^10^ and B.1.617.2 (Delta)^11^, which has led to impairment of the efficacy of the neutralizing antibody treatments^12–14^ and also compromised the effects of approved vaccines^15,16^.

Omicron BA.1 (B.1.1.529.1) was first identified in South Africa in November 2021^17^ and soon designated variants of concern (VOC) by WHO (https://www.who.int/en/activities/tracking-SARS-CoV-2-variants/). Omicron spread rapidly since its appearance^18,19^ and became the globally dominant strain within a few weeks. Omicron harbors more than 30 mutations in the spike protein, including 15 mutations in the receptor-binding domain (RBD)^20,21^. The potency of many nmAbs has been impaired against Omicron^21–25^. Specifically, Liu et al. showed that the activity of 17 of 19 tested nmAbs against Omicron BA.1 was either completely knocked out or severely impaired^23^. Moreover, Planas et al. demonstrated that Omicron BA.1 was totally or partially resistant to neutralization by 9 nmAbs which are clinically approved or in development; only sotrovimab exhibited a rather similar activity against Delta and Omicron BA.1 with IC_50_ values of 325 ng/mL and 917 ng/mL, respectively^25^. Soon after the emergence of BA.1, a number of Omicron subvariants have successively emerged, including BA.1.1, BA.2, BA.2.12.1, BA.2.13, BA.3, BA.4, and BA.5. Among them, BA.2 and BA.5 have recently become the dominant strains worldwide^26^.

The use of cocktail treatment, involving more than one non-competing nmAbs, may lead to increased efficacy and better resistance to viral escape than monotherapy, whilst posing additional challenges for clinical translation. Multiform bispecific antibodies against SARS-CoV-2 have been developed, including those to Omicron and Omicron subvariants^27–31^, and biparatopic antibodies are special bispecifics that are able to simultaneously target two different epitopes of one antigen, thus may possess even higher binding affinity and efficacy compared to ordinary bispecifics.

Here, we report the isolation and characterization of several highly potent neutralizing mAbs against SARS-CoV-2 variants from human antibody transgenic mice. A biparatopic antibody BA7125/7208 combining non-competing mAbs BA7208 and BA7125 was able to potently neutralize all tested SARS-CoV-2 variants including BA.5. Cryo-electron microscopy (cryo-EM) analysis showed that BA7208 recognizes highly conserved residues. Additionally, BA7125/7208 was shown to protect K18-hACE2-transgenic mice against BA.1 and BA.2 infections through various administration routes.

## Results

### Identification of potential antibodies against a broader array of SARS-CoV-2 variants through sequential immunization and screening

Antibodies were isolated using immunized human antibody transgenic mice BA-huMab^®^-derived phage-display libraries as previously described^32^ (Supplementary Fig. S1). To obtain antibodies with potency against a broader array of variants, sequential immunization and/or sequential screening were performed. Each mouse was immunized for 4–5 rounds with the RBDs or trimeric Spike ectodomain proteins in the pre-fusion conformation of Beta, Gamma, Kappa, or Delta variants (for details, please see Materials and methods and Table 1).

**Table 1:**
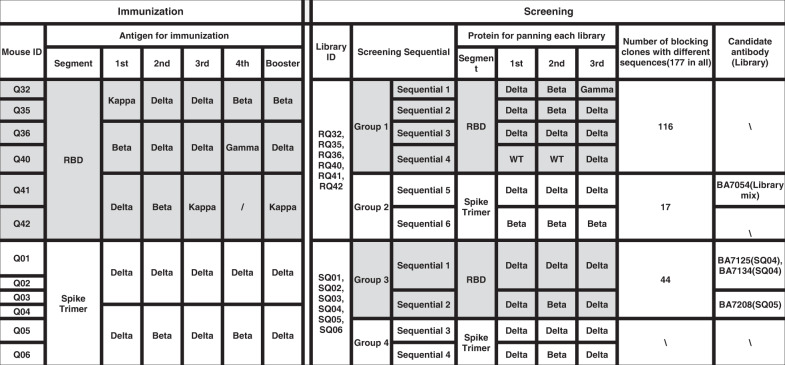
Sequential immunization and sequential screening regimen.

Spike or RBD proteins from diverse variants were used as antigens to elicit anti-Spike antibodies. Different Spike or RBD proteins were used as baits for each round of screening to capture cross-reactive anti-Spike antibodies from phage libraries. Blocking clones mean the clones which can secret scFvs to block the ACE2 binding to Spike protein.

**Fig. 1:**
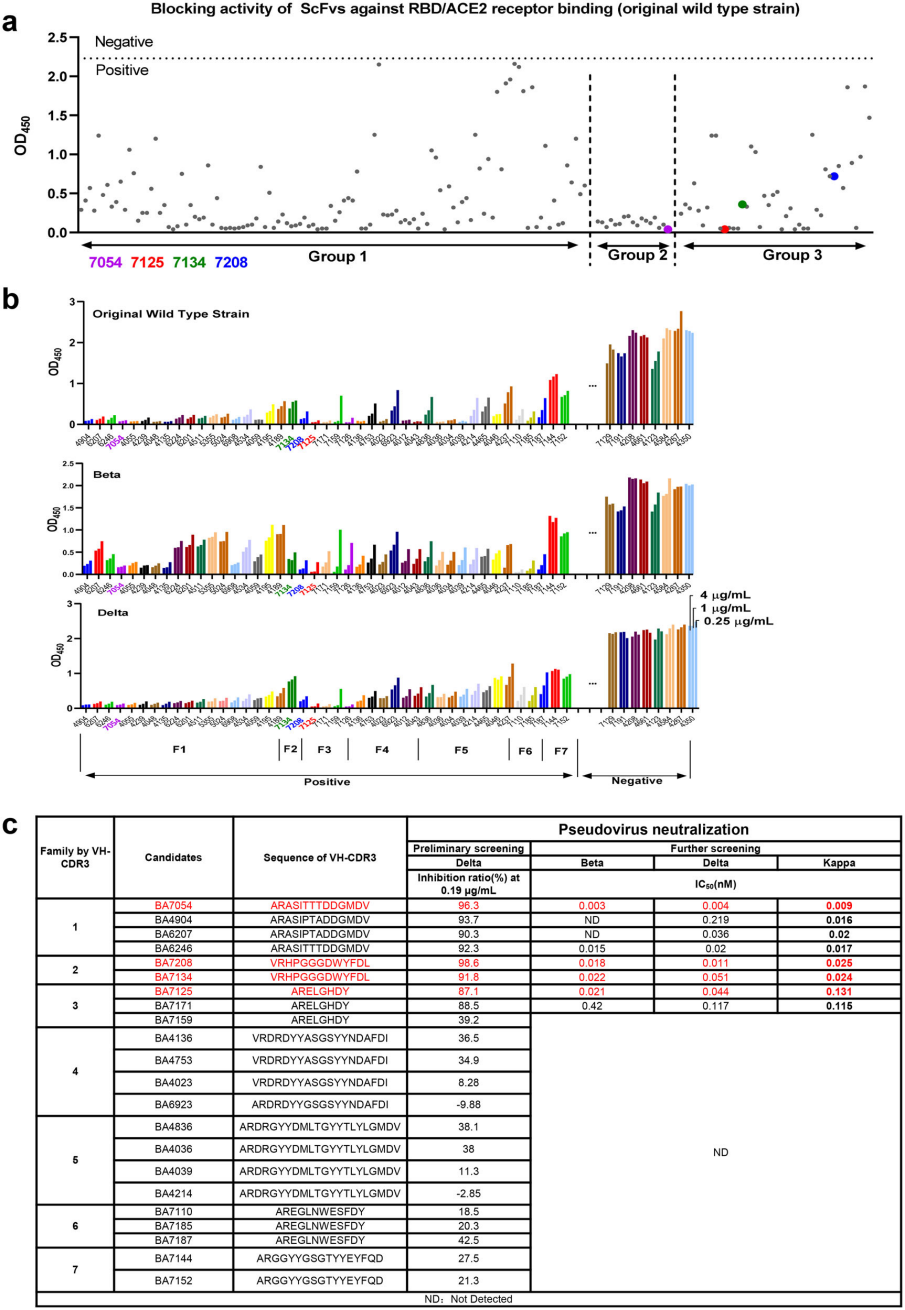
Identification of potential antibodies against a broader array of variants. **a** RBD/ACE2 receptor blocking activity of the 177 ScFvs. RBD is from the original wild-type (WT) strain. Group 1: immunization with RBD protein and screening with RBD protein; Group 2: immunization with RBD protein and screening with Spike protein; Group 3: immunization with Spike protein and screening with RBD protein. OD_450_ < 2.3 in the ELISA-based receptor-binding inhibition assay was defined as “positive” and OD_450_ > 2.3 was defined as “negative”. **b** Blocking ACE2 receptor binding to RBDs of original WT strain, beta and delta strain by the 177 mAbs. mAbs were diluted as 0.25, 1, 4 μg/mL. These mAbs were grouped into seven families by heavy chain CDR3 sequence homology. **c** Neutralization activity of the 22 mAbs in 0.19 μg/mL using vesicular stomatitis virus (VSV) pseudotyped with Delta Spike protein was shown for preliminary screening. 8 mAbs were selected to test neutralization activities using VSV pseudotyped with Delta, Beta, or Kappa Spike protein for further screening, and IC_50_ values were shown.

Antibody phage library was constructed for each immunized mouse. Sequential screening strategy was used to enrich cross-reactive antibodies against the Spike proteins from various SARS-CoV-2 variants (Table 1). Six sequential screenings, using four RBD proteins (group 1) and two Spike proteins (group 2), were performed for each RBD-immunized library. Following the enzyme-linked immunosorbent assay (ELISA)-based receptor-binding inhibition assay, 116 single-chain variable fragments (ScFvs) and 17 ScFv clones were shown to block RBD/ACE2 binding. Four sequential screenings, using two RBD proteins (group 3) and two Spike proteins (group 4), were performed for each Spike-immunized library. 44 ScFvs with blocking activities were isolated from the RBD screening but none from Spike screening. The ACE2-blocking activity of these 177 ScFvs is showed in Fig. 1a.

The identified ScFvs were converted to IgG1 antibodies for further in vitro blocking and pseudovirus neutralization evaluation. Among the 177 mAbs, a total of 43 mAbs were shown to broadly block the binding of RBD variants to ACE2 with an OD_450_ < 1.4 in an ELISA-based receptor-binding inhibition assay, including original wild-type (WT), Delta and Beta strains (Fig. 1b). These 43 mAbs were grouped into seven families by heavy chain CDR3 sequence homology. Two to four promising candidates in each family, giving a total of 22 mAbs, were picked for preliminary pseudovirus neutralization evaluation (Fig. 1c). Antibodies from family 1, 2, 3 demonstrated remarkable neutralizing activity with inhibition ratio > 80% but the mAbs from family 4, 5, 6, 7 did not demonstrate blocking activity. The positive clones from family 1, 2, and 3 were selected for further pseudovirus neutralization evaluation. The top four candidates showing the best breath and potency were BA7054, BA7208, BA7134, and BA7125 (Fig. 1c) which belong to the IGHV3-30, IGHV5-51, IGHV5-51, and IGHV4-34 antibody lineage, respectively. Three of them were isolated from mice immunized with the trimeric Spike pre-fusion proteins of Beta and Delta variants, which demonstrates the superiority of spike pre-fusion trimers as antigens.

### BA7125, BA7054, BA7208 and BA7134 cross-neutralized diverse SARS-CoV-2 variants

**Fig. 2:**
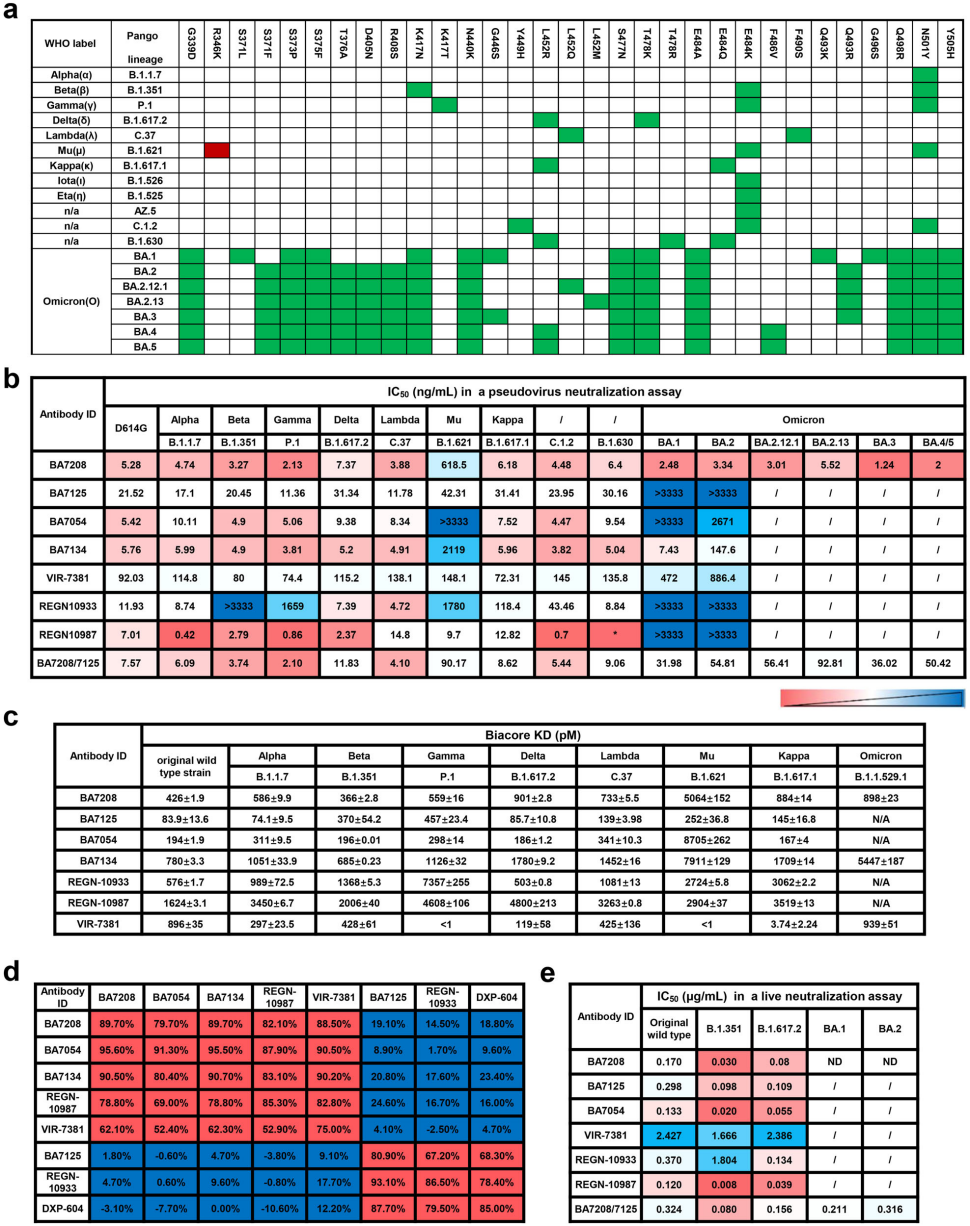
MAbs binding and neutralization of diverse SARS-CoV-2 variants. **a** Key substitutions in RBD in 19 previous and current variants. **b** Neutralization activity of the indicated antibodies against the SARS-CoV-2 variants examined in human Huh7 cells using a pseudovirus system. “/”: These antibodies show low neutralization activity against BA.1 and BA.2, therefore no further research against other Omicron variants were performed. **c** *K*_D_ values of the indicated antibodies to RBD of various variants examined by SPR. Data were collected from two biological replicates and represented as means ± SD. **d** Competitive binding of the antibodies in BLI-based competitive binding assay. Inhibitory rate was shown as a percentage. Antibody A displaying > 50% inhibitory rate against antibody B means antibody A strongly competes with antibody B to bind the RBD. **e** Neutralizing titer IC_50_ values of the indicated antibodies against the authentic SARS-CoV-2 variants in FRNT assays. “/”: These antibodies show low neutralization activity. ND not detected. Data were collected from two biological replicates and represented as means ± SD.

Mutated RBD amino acid substitutions for 19 SARS-CoV-2 variants are shown in Fig. 2a. We examined if the four antibodies could efficiently neutralize these variants, using the approved antibodies Vir-7381, REGN10933, and REGN10987 as control. Variants containing only one E484K mutation in RBD, including Lota, Eta, and AZ.5, were not evaluated. The in vitro neutralization abilities of each antibody against the remaining 16 variants and D614G parental variant were examined in human Huh7 cells using VSV pseudovirus system expressing the respective Spike protein (Fig. 2b and Supplementary Fig. S2). BA7208 showed the ability to efficiently neutralize 15 out of 16 variants except Mu variant, with IC_50_ values from 1.24 ng/mL to 7.37 ng/mL. It maintained high potency against Omicron BA.1–BA.5 with an IC_50_ value from 1.24 ng/mL to 5.52 ng/mL. The impaired activity of BA7208 by Mu variant is probably attributed to the R346K RBD mutation (Fig. 2b). BA7125 was able to neutralize all variants with an IC_50_ value from 11.36 ng/mL to 42.31 ng/mL but Omicron variants. BA7054 was able to neutralize 9 variants with an IC_50_ value from 4.47 ng/mL to 10.11 ng/mL but showed no activity against Mu and Omicron variants. BA7134 was able to neutralize 11 variants with an IC_50_ value from 3.81 ng/mL to 147.60 ng/mL but its activity against Mu was completely knocked out.

The binding affinity of these antibodies to the RBD of various variants was examined by surface plasmon resonance (SPR) (Fig. 2c). BA7208 was able to bind tightly to 8/9 RBDs except the Mu RBD, with a *K*_D_ value from 366 pM to 901 pM. BA7208 and BA7134 were able to bind Omicron BA.1 RBD with a *K*_D_ of 898 pM and 5447 pM, respectively. BA7125 showed strong binding affinity against 8 out of 9 RBDs with a *K*_D_ value from 74.1 pM to 457 pM, but negligible binding to Omicron BA.1 RBD. BA7054 demonstrated tight binding for 7/9 RBDs with a *K*_D_ ranging from 167 pM to 341 pM; its affinity for Mu RBD was much lower (*K*_D_ = 8705 pM), and binding to Omicron BA.1 RBD was completely impaired. Comparable results were obtained through binding free energy calculations (Supplementary Table S1).

Analysis of blocking activity by ELISA-based receptor-binding inhibition assay showed that these four antibodies efficiently blocked binding of Beta and Delta RBD to ACE2, but only BA7208 demonstrated remarkable blocking activity for Omicron RBD (Supplementary Fig. S3). Given that these nmAb candidates were capable of broadly neutralizing various SARS-CoV-2 variants, we compared their preliminary binding epitopes to Delta RBD with VIR-7381, REGN10933, and REGN10987 using a Bio-layer interferometry (BLI) based competition assay. The data showed BA7208, BA7054, VIR-7381, and REGN10987 competed with each other, suggesting their epitopes were close to each other; and BA7054 and BA7208 may also bind an epitope at the margin of ACE2-binding region like VIR-7381 and REGN10987. BA7208 and BA7125 did not compete with each other, indicating their distinct epitopes (Fig. 2d).

### Biparatopic antibody BA7208/7125 effectively neutralized all tested SARS-CoV-2 variants including Omicron BA.1-BA.5

Given that binding epitope of BA7208 are distinct from BA7125, we constructed a bispecific antibody BA7208/7125 with one arm from the Fab of BA7125 and the other arm from the ScFv of BA7208 (Supplementary Fig. S4a). The knobs-into-holes technology was introduced into the bispecific to promote heterodimerization, involving engineering CH3 domains to create either a “knob” or a “hole” in each heavy chain, which has been widely applied^33^. We compared the in vitro neutralization abilities of BA7208/7125 with BA7208 or BA7125 against the 18 SARS-CoV-2 variants using the VSV pseudovirus neutralization assay (Fig. 2b and Supplementary Fig. S4b). BA7208/7125 exhibited increased breadth and was able to neutralize all tested variants with an IC_50_ value from 2.10 ng/mL to 92.81 ng/mL. Both BA7125 and BA7208 contribute to the increased breadth. No one variant tested was observed to escape from neutralization by this bispecific antibody. In vitro neutralization activities of BA7208, BA7125, BA7054, and BA7208/7125 against the authentic virus were examined using the focus reduction forming assays (FRNT). It was shown that all four antibodies were potently neutralizing against WT, B.1.351 and B.1.617.2 variants (Fig. 2e and Supplementary Fig. S4c). BA7208/7125 was able to strongly neutralize authentic SARS-CoV-2 variants Omicron BA.1 and BA.2 with IC_50_ values from 211 to 316 ng/mL.

Bispecific may simultaneously bind two different epitopes of one RBD and this bivalency would provide higher binding and naturalization activities. Firstly, we examined whether BA7208/BA7125 demonstrated higher affinities against Omicron BA.1, Mu, and Delta RBD than the single mAbs using the SPR technology (Supplementary Fig. S5). Binding of BA7208/7125 to Omicron BA.1 RBD protein was ~3.7-fold higher than that of BA7208 (*K*_D_ = 150 pM vs 557 pM). Its binding to the Mu RBD protein was ~3.6-fold tighter than that of BA7125 (*K*_D_ = 59.0 pM vs 215 pM). These results suggested that BA7208/7125 may simultaneously bind to two different epitopes of one RBD and so it was termed as biparatopic antibody.

### Cryo-EM analysis of the trimeric SARS-CoV-2 Spike in complex with BA7208-Fab, BA7125-Fab, and BA7054-Fab

**Fig. 3:**
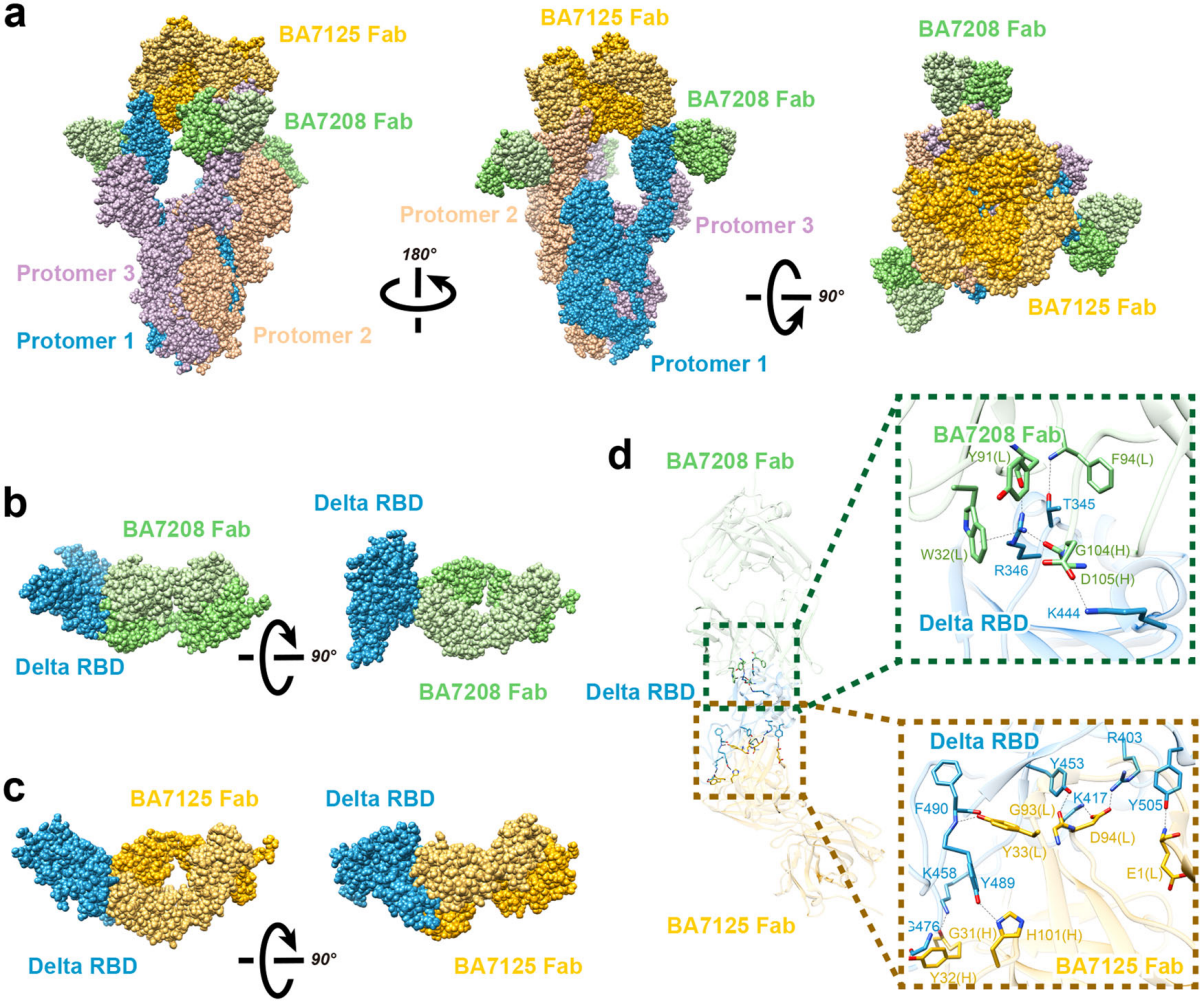
Cryo-EM of Delta Spike Trimer with BA7208-Fab/BA7125-Fab. **a** The complex of three BA7208-Fabs (green) and three BA7125-Fabs (yellow) with Delta Spike Trimer (dodger blue, plum, and rosy brown). **b** The complex of BA7208-Fab (light green, Fab light chain; dark green, Fab heavy chain) with Delta Spike-RBD (dodger blue). **c** The complex of BA7125-Fab (light yellow, Fab light chain; dark yellow, Fab heavy chain) with Delta Spike-RBD (dodger blue). **d** Zoomed-in views of Fabs binding sites on Delta RBD. Side chain of residues that form the hydrogen bonds, salt bridge, and one cation-π interaction are displayed.

To gain insight of the structural basis for their broad-spectrum properties and the neutralizing mechanism of BA7208/7125, we determined the complex structure of a stabilized pre-fusion trimeric Delta Spike with BA7125-Fab and BA7208-Fab using cryo-EM. Details of sample preparation, data collection, and EM analysis are in the methods and extended data (Supplementary Fig. S6; Table S2). The resolution of the overall structure was 3.08 Å, in which all three RBDs of the Spike were in the “up” conformation (Fig. 3a). The resolution of the RBD/Fab interface was improved to 2.98 Å by focusing refinement on one RBD/Fab sub-complex (Fig. 3b, c). Analysis of the structure showed that the Spike trimer was bound by three BA7208-Fabs protruding outward while binding to the RBDs and three BA7125-Fabs leaning on each other along the central axis of Delta Spike. Consistent with the BLI results, BA7208 and BA7125 bound different regions on RBD. The BA7208-Fab bound an area at the palm region of the RBD. We have identified four hydrogen bonds, one salt bridge, and one cation-π interaction formed between resides from the BA7208-Fab and Delta-RBD (Fig. 3d and Supplementary Table S3). The hydrogen bonds include T345-F94, R346-Y91, R346-G104, and K444-D105 (core residues are from the RBD and hind residues are from the BA7208-Fab). Residue K444 on RBD and D105 on 7208-Fab formed a salt bridge, while a cation from positively charged residue R346 interacted with an aromatic ring from residue W32 on the BA7208-Fab to form a cation-π interaction.

The BA7125-Fab bound an area at the thumb region of the RBD. At binding site of BA7125-Fab, eight hydrogen bonds and two salt bridges were formed with Delta RBD (Fig. 3d and Supplementary Table S4). The hydrogen bonds formed between R403-D94, L417-D94, Y453-G93, K458-G31, G476-Y32, Y489-H101, F490-Y33, and Y505-E1. RBD residues R403 and K417 form two salt bridges with D94 in the BA7125-Fab. The interaction buried a total of 693 Å^2^ surface area at BA7208-Fab/RBD interface, and 1099.4 Å^2^ between the 7125-Fab and the Delta RBD.

In addition, the structure of the Delta Spike in complex with BA7054-Fab and BA7208-Fab was also solved at a consensus map of 3.21 Å (Supplementary Fig. S7; Table S5). Similar to the Delta-Spike/BA7208-Fab/BA7125-Fab complex, all three RBDs in this complex were in the “up” conformation with three BA7054-Fabs protruding outward and three BA7125-Fabs along the central axis of Delta S (Supplementary Fig. S8a). The resolution of the RBD/Fab interface was increased to 3.18 Å by focusing refinement on one RBD/Fab sub-complex (Supplementary Fig. S8b, c). At the Delta-RBD/BA7054-Fab interface, we identified six hydrogen bonds and two salt bridges formed between resides D54, N57, A91, T103, D105, and D106 from the BA7054-Fab and residues R346, N440, K444 from the Delta-RBD (Supplementary Fig. S8d; Table S6).

**Fig. 4:**
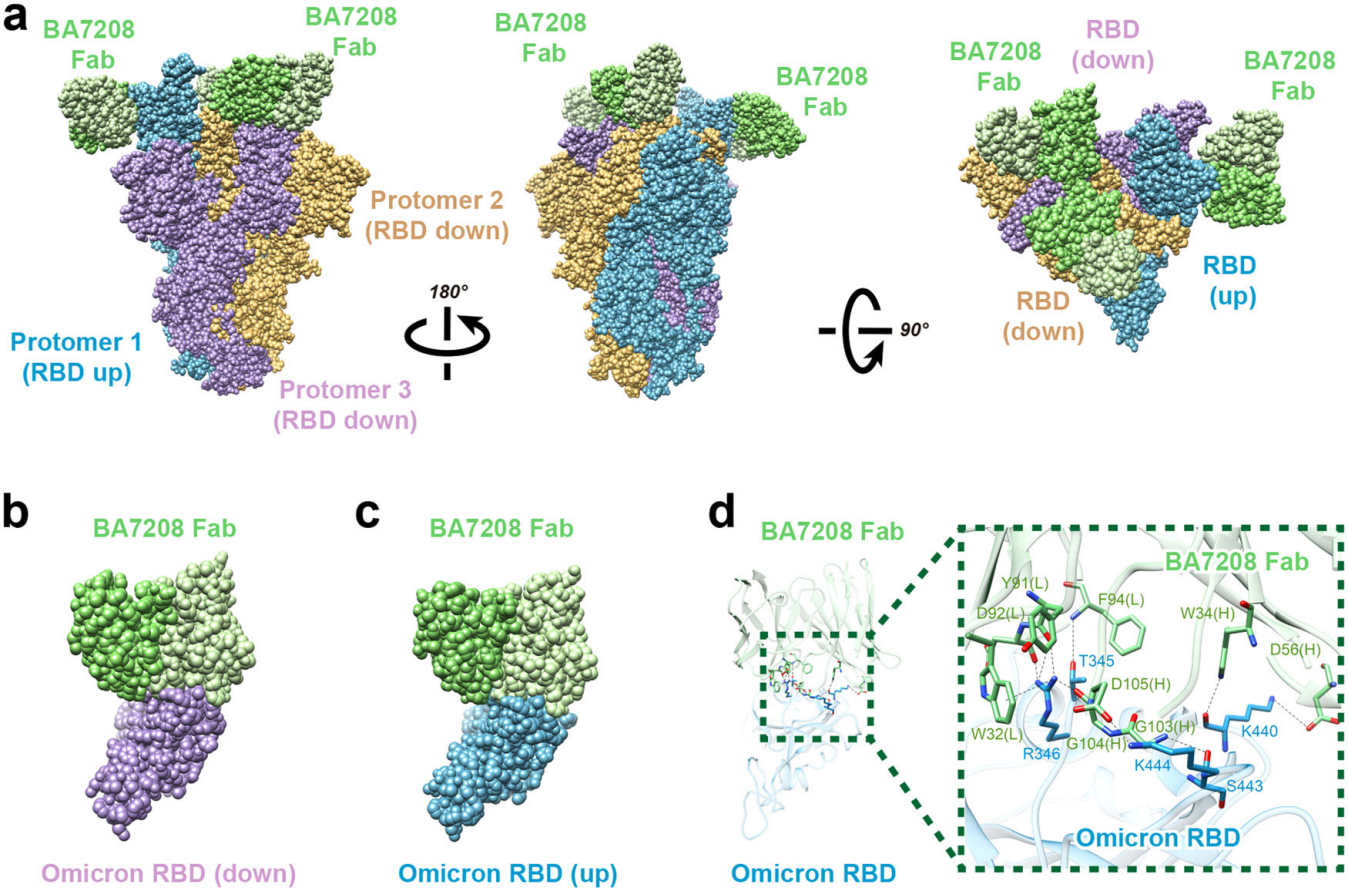
Cryo-EM of Omicron Spike Trimer with BA7208-Fab. **a** The complex of three BA7208-Fabs (green) with Omicron Spike Trimer (dodger blue, plum, and rosy brown). **b** The complex of BA7208-Fab (light green, Fab light chain; dark green, Fab heavy chain) with Omicron Spike-RBD in the down conformation (plum). **c** The complex of BA7208-Fab with Omicron Spike-RBD in the up conformation (dodger blue). **d** Zoomed-in views of 7208-Fab binding site on Omicron RBD. Side chain of residues that form the hydrogen bonds, salt bridge, and one cation-π interaction are displayed.

To characterize the antibody binding diversity between Delta and Omicron variants, we determined the structure of trimeric Omicron Spike in complex with BA7208-Fabs at 2.62 Å resolution (Supplementary Table S7). Interestingly, here only one RBD was in the “up” conformation, and the other two RBDs were “down” (Fig. 4a); two local structures of BA7208-Fab binding with up-RBD and down-RBD are identical (Fig. 4b, c). At the interface between Omicron Spike-RBD and BA7208-Fab, we identified eight hydrogen bonds, three salt bridges, and one cation-π interactions (Fig. 4d and Supplementary Table S8). The hydrogen bonds formed between T345-F94, R346-Y91, R346-D92, R346-G104, K440-W34, K440-D56, S443-G103, and K444-D105. Three salt bridges included R346-D92, K440-D56, and K444-D105. The cation-π interaction included an aromatic ring residue W32 from BA7208-Fab and positively charged resides R346 from Omicron-RBD. R346 played a key role in the interaction between Spike-RBD and BA7208 confirmed that the R346K mutation in Mu RBD was responsible for the impaired activity of BA7208 (Fig. 2a). Further structural analysis revealed that 14 out of 15 RBD mutations on the Omicron BA.1 RBD did not directly interact with BA7208-Fab; only N440K formed hydrogen bonds with W34 and D56 from the Fab heavy chain (Fig. 4d and Supplementary Fig. S9). None of the additional three RBD mutations from Omicron BA.2 (T376A, D405N, and R408S) were found to be located at the BA7208 binding sites.

**Fig. 5:**
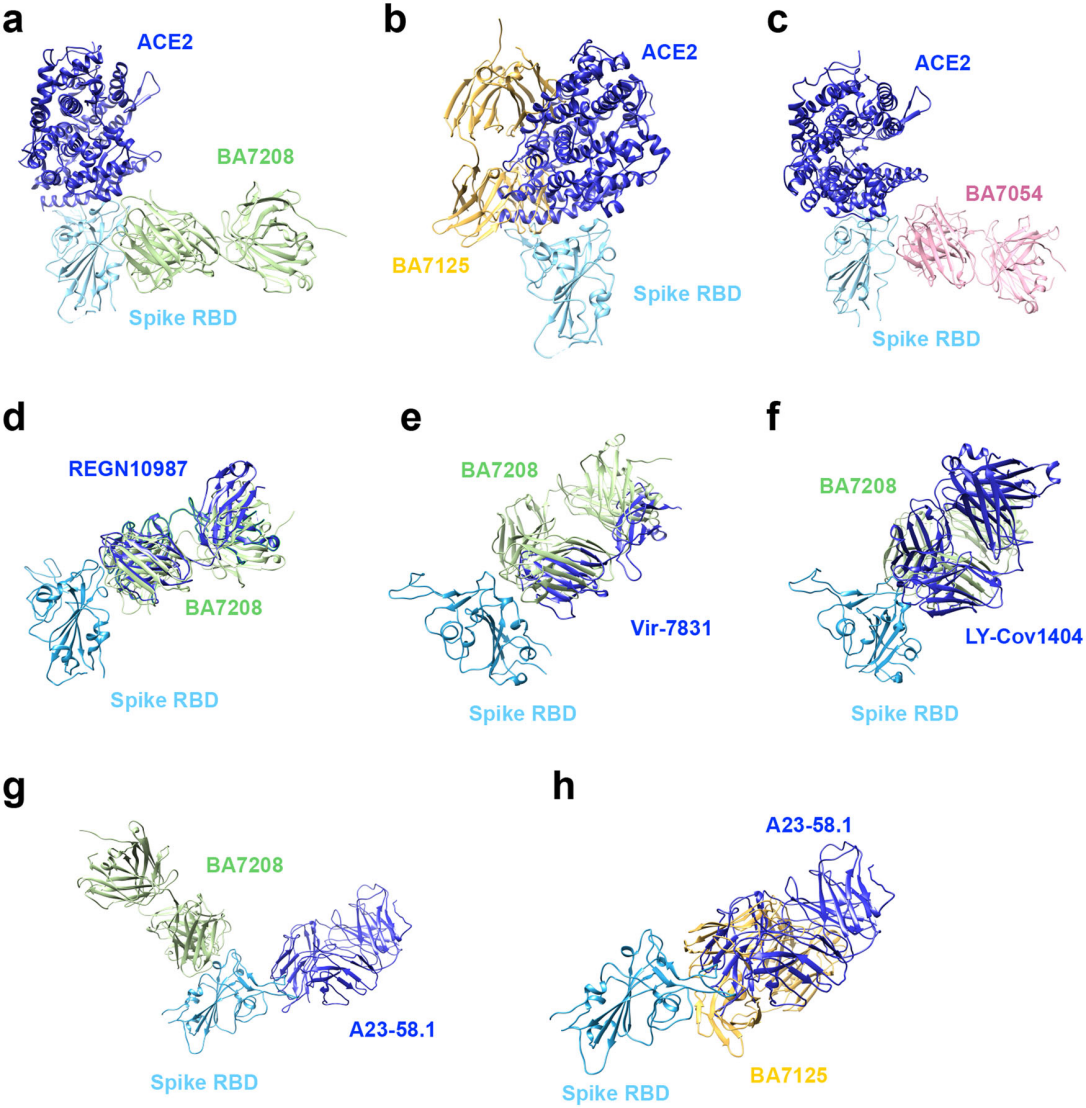
BA7208/BA7125 structure superimpose analysis. **a** The complex of RBD (cyan) and BA7208-Fab (green) is superimposed with complex of RBD (cyan) and ACE2 (blue). **b** The complex of RBD (cyan) and BA7125-Fab (yellow) is superimposed with complex of RBD (cyan) and ACE2 (blue). **c** The complex of RBD (cyan) and BA7054-Fab (pink) is superimposed with complex of RBD (cyan) and ACE2 (blue). **d**–**g** The complex of RBD (cyan) and BA7208-Fab (green) is superimpose with complex of RBD (cyan) and antibodies (blue) REGN10987, Vir-7381, LY-Cov1404, and A23-58.1, respectively. **h** The complex of RBD (cyan) and BA7125-Fab (yellow) is superimposed with complex of RBD (cyan) and A23-58.1 antibody (blue).

To investigate the mechanism for neutralization, we superimposed the structure of Spike-RBD/ACE2 complex (PDB ID: 6VW1) with that of RBD/BA7208-Fab, RBD/BA7125-Fab and RBD/BA7054-Fab (Fig. 5a–c). Unlike BA7125-Fab which was shown to clash with ACE2 binding, neither BA7208-Fab nor BA7054-Fab was able to directly block ACE2 binding to Spike-RBD. Therefore, the mechanism through which BA7208 and BA7054 exhibited remarkable blocking activity in the IgG format remains elusive (Supplementary Fig. S4), probably through a steric interference.

Next, we compared the binding site of BA7208-Fab with that of REGN10987 (PDB ID: 6XDG) and VIR-7831 (PDB ID: 7R6X) in Spike-RBD (Fig. 5d, e). Consistent with the BLI competition results, binding mode of BA7208-Fab was similar to REGN10987 and VIR-7831 (Fig. 2d). Then we performed the structural comparisons of RBD/BA7208-Fab with two broadly and potently neutralizing antibodies, RBD/LY-COv1404 (PDB ID: 7MMO) and RBD/A23-58.1(PDB ID: 7LRS)^34,35^. Binding mode of BA7208 was similar to that of LY-COv1404 but distinct from A23-58.1 (Fig. 5f, g). In contrast, BA7125 and A23-58.1 shared a similar binding mode (Fig. 5h). According to the four classes of antibodies targeting the RBD^26,36^, BA7208, BA7125, and BA7054 are categorized into class III (binding RBD up or down, with no overlap with ACE2 binding site), class I (binding RBD up only, ACE2-blocking) and class II (binding RBD up or down, ACE2-blocking), respectively.

Structural analyses revealed that breadth of BA7208, BA7125, and BA7054 was mediated by targeting the regions of the RBD that are not overlapping the mutational sites of most current variants (Fig. 2a). The smaller binding area on the RBD bound with BA7208 (Fig. 4d) also contributed to its broad-spectrum by reducing the risk of being affected by RBD mutations. The observation that the Fab of BA7125 and BA7208 were able to bind simultaneously to the same RBD provided the structural basis for the rational design of biparatopic antibody BA7208/7125.

### BA7208 and BA7208/7125 protected hACE2-transgenic and WT mice from SARS-CoV-2 infections via diverse administration routes

**Fig. 6:**
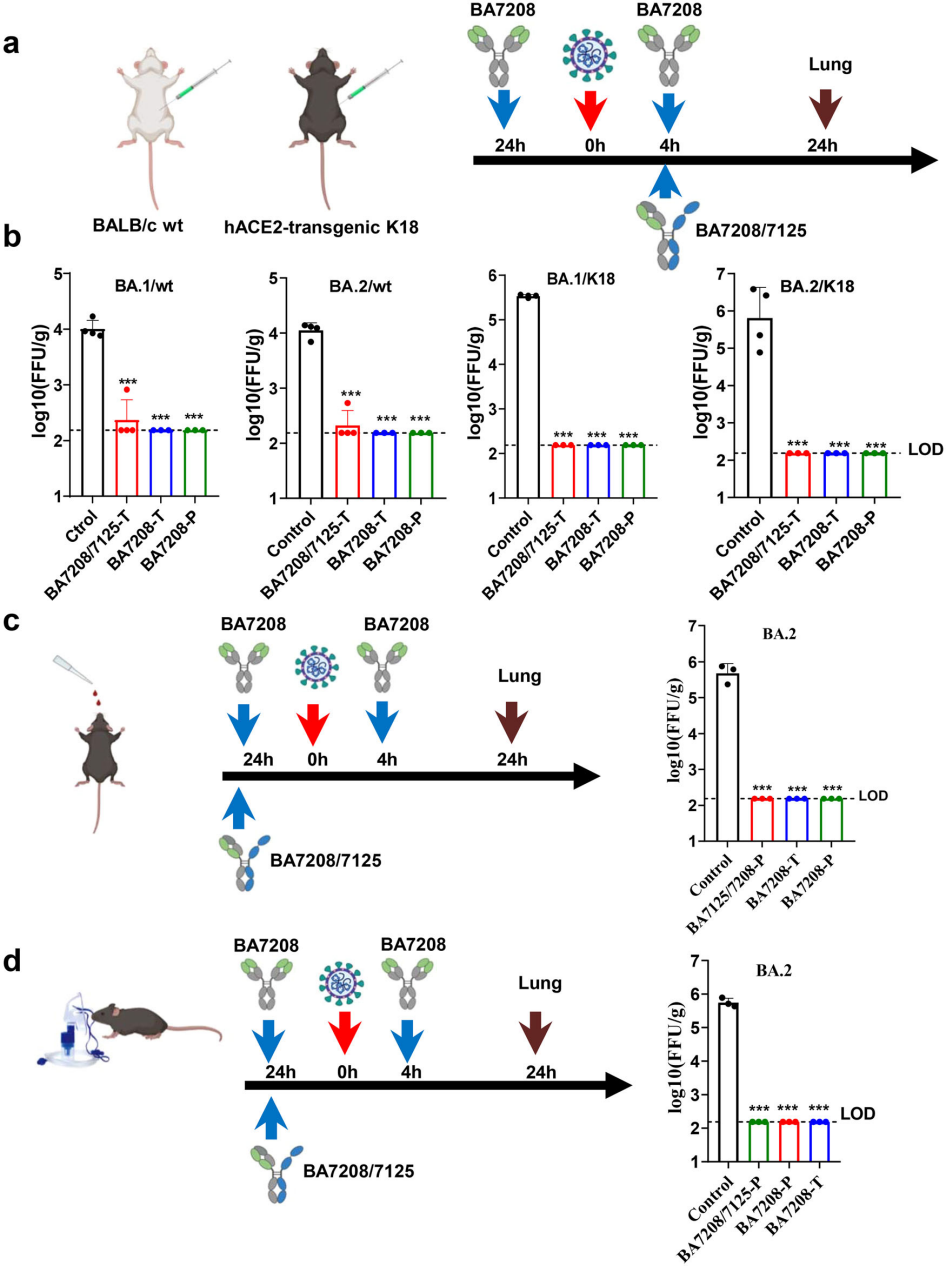
BA72018/7125 and BA7208 protected mice from SARS-CoV-2 variant infection. **a** BA72018/7125 and BA7208 were administrated to K18-hACE2-transgenic and BALB/c (wt) mice (6–8 weeks old, female) intraperitoneally at a dose of 10 mg/kg 24 h before (prophylactic, abbreviation P) or 4 h after (therapeutic, abbreviation T) SARS-CoV-2 Omicron BA.1 or BA.2 infection (1 × 10^5^ FFU, intranasally). PBS was administrated as a negative control. **b** Lungs were harvested for viral titers at 1 day post infection (dpi) for prophylactic and therapeutic group (*n* = 3 or 4 mice per group) by FFA method. LOD limit of detection, g gram,. wt BALB/c wt mouse, K18 K18-hACE2-transgenic mouse. **c** BA7208 and BA72018/7125 were administered via intranasal at a dose of 1 mg/kg 24 h before (prophylactic, abbreviation P) or 4 h after (therapeutic, abbreviation T) SARS-CoV-2 Omicron BA.2 infection (1 × 10^5^ FFU, intranasally). Lungs were harvested for viral titers at 1 dpi. (*n* = 3 or 4 mice per group). **d** BA72018/7125 and BA7208 were administered via aerosol inhalation at a dose of 3 mg/kg 24 h before (prophylactic, abbreviation P) or 4 h after (therapeutic, abbreviation T) SARS-CoV-2 Omicron BA.2 infection (1 × 10^5^ FFU, intranasally). Lungs were harvested for viral titers at 1 dpi. (*n* = 3 or 4 mice per group).

Biparatopic antibody BA7208/7125 and mAb BA7208 were selected for in vivo validation in light of their breath and strong potency against different SARS-CoV-2 variants. The prophylactic and therapeutic efficacies of BA7208/7125 and BA7208 were evaluated using two mouse models as previously described^37,38^, namely hACE2-transgenic mouse and BALB/c WT mouse. Mice were infected intranasally with authentic SARS-CoV-2 Omicron BA.1 or BA.2. Three to four mice per group were treated with BA7208 or BA7208/7125 at a dose of 10 mg/kg intraperitoneally 24 h before or 4 h after SARS-CoV-2 infection (Fig. 6a). As previously reported, no or only mild clinical signs of disease were observed after SARS-CoV-2 Omicron viral challenge but viral particles were detected in lung samples^37^. Lungs were harvested at day 1 post infection for quantification of viral titers by focus forming assay (FFA) (Fig. 6b). Mice in both prophylactic and therapeutic groups showed a significant reduction in lung viral titers. In the WT mouse model, lung viral titers reduced 2-log in both the prophylactic and therapeutic groups. In the hACE2-transgenic mouse model, both prophylactic and therapeutic groups showed greater degree of reduction in lung viral titers (3-log) compared with the control group; the infectious viral titers were below the limit of detection (LOD) in both groups.

Since the respiratory tract is the most important portal of entry for SARS-CoV-2, respiratory administration directly to the airways could be an effective way to deliver the neutralizing antibodies. To test this hypothesis, hACE2-transgenic mice were treated with BA7208 or BA7208/7125 intranasally at 1 mg/kg or through aerosol inhalation at 3 mg/kg 24 h before or 4 h after SARS-CoV-2 challenge (Fig. 6c, d). Analysis of viral load in the post-mortem lungs was performed 24 h after viral challenge through the FFA. No live viral particle was detected in either the prophylactic or therapeutic group, whereas all animals in the control group revealed high viral titers in the lungs.

### BA7208/7125 demonstrated a long half-life comparable with BA7208 or BA7125 in mice

The pharmacokinetics of BA7208/7125, BA7208, and BA7125 were studied in mice. To this, a single dose of the antibody was intravenously administered to the C57BL/6 mice (*n* = 3) at 10 mg/kg. ELISA was used to determine the antibody concentration in serum. BA7208, BA7125, and BA7208/7125 showed comparable, long half-lives and AUC_(0–*t*)_, with a terminal half-life (*t*_1/2_, *λ*_z_) of ~94 h, 128 h, and 100 h, respectively; and an AUC_(0–*t*)_ of ~8577 h*μg/mL, 9991 h*μg/mL, and 7294 h*μg/mL, respectively, (Supplementary Fig. S10; Table S9).

## Discussion

The continuous emergence of highly immune-invasive SARS-CoV-2 variants has resulted in impairment of the effectiveness of available monoclonal antibodies. To this, FDA has halted the EUA of most therapeutic antibodies, including LY-CoV555, CB6, REGN10987 and REGN10933. Hence, there is an urgent need to develop broad-spectrum and potently neutralizing antibodies to combat the ongoing SARS-CoV-2 pandemic. Here, by cross-immunizing transgenic mice engineered to express human antibodies with Beta and Delta Spike proteins, we isolated a number of potently neutralizing antibodies among which BA7208 and BA7125 also showed broadly neutralizing activities. Structural analysis through cryo-EM confirmed results from a BLI-based competition assay that BA7208 and BA7125 bind non-overlapping epitopes on the Spike-RBD. It was shown that BA7208 has a footprint distal to core ACE2-binding sites in RBD protein, while BA7125 binds to the palm region of the RBD.

BA7208 and BA7125 were engineered into a bispecific antibody called BA7208/7125 through the knobs-into-holes technology. BA7208/7125 was able to potently neutralize all tested SARS-CoV-2 variants including BA.5. In vivo studies showed that administration of BA7208/7125 via respiratory or intraperitoneal administration demonstrated potent therapeutic and prophylactic efficacy against Omicron BA.1 and BA.2 in hACE2-transgenic and WT mice.

This ‘original antigenic sin’ phenomenon observed in influenza has raised some concerns about whether boosting Omicron-based vaccine following previous immunizations with the wild strain-based vaccine^39,40^. Antigenic distance among different SARS-CoV-2 variants could explain how the efficacy of vaccines could be influenced by the difference or relatedness of prior vaccinations. This study using sequential immunization regimen supports the rationale that boosting Omicron-based vaccines following previous immunizations with the wild strain-based vaccine would selectively induce the immune responses cross-reactive with both the wild strain and Omicron^41–43^. Given that the respiratory tract is the most important site for viral entry, transmission, and spread, we compared the performance of different administration routes including intraperitoneal, intranasal and aerosol inhalation. The results indicated that delivery of antibodies BA7208 and BA7208/7125 through multiple administration routes protected mice from SARS-CoV-2 infection in prophylactic and therapeutic settings, which is similar to single-domain antibody administration^44^. Mice in both prevention and treatment groups showed accelerated viral clearance in the lungs, suggesting that these antibodies can be efficiently delivered and treat pulmonary disease via diverse routes.

This study also has some limitations. First, neutralization assays were performed with a limited range of SARS-CoV-2 authentic viruses, including WT, Beta, Delta, Omicron BA.1 and BA.2; neutralization assays against BA.5 authentic viruses are to be performed in the future. Second, in line with previous studies, due to invisible or only mild clinical signs in mice with the Omicron strains, we have not tested the lung histology in these mice^37^. The antibody doses used in the animal experiments through intranasal and aerosol inhalation administrations were designed based on previous studies^44^, with limited guidance in clinical trials.

In summary, we report the isolation of potently and broadly neutralizing mAbs from transgenic mice engineered to express human antibodies, which could be a quicker and more effective way to generate fully human antibodies against different antigens. Biparatopic antibody BA7208/7125 effectively neutralizes all tested SARS-CoV-2 variants and could be resistant to the ever-evolving variants. BA7208/7125 was protective against Omicron BA.1 and BA.2 in hACE2-transgenic and WT mice not only through the intraperitoneal delivery route but also through intranasal and aerosol inhalation administrations, thus making it a promising candidate as an intervention against COVID-19.

## Materials and methods

### Ethical statement

All animal experiments complied with relevant ethical regulations regarding animal research. Immunization and pharmacokinetics study procedures in mice were approved by the Institutional Animal Care Committee of Boan Biotech and the Approval Numbers are 2021-TS0001-20 and 2021-TS0001-37, respectively. The animal study was reviewed and approved by the Institutional Animal Care and Use Committees of the Guangzhou Medical University (2021-239).

### Viruses and mice

The K18-hACE2-transgenic mice were purchased from Gem Pharmatech Co., Ltd, and BALB/c mice were purchased from Jinan Pengyue Experimental Animal Breeding Co. LTD. The SARS-CoV-2 variants including Alpha (B.1.1.7), Beta (B.1.351), and Omicron (BA.1 and BA.2) were isolated from COVID-19 patients in Guangdong China. The SARS-CoV-2 Delta (B.1.617.2) strain was presented by Guangdong Provincial Center for Disease Control and Prevention, China. Focus-forming assay (FFA) was used to quantify the virus titer. Experiments related to authentic SARS-CoV-2 were conducted in Guangzhou Customs District Technology Center BSL-3 Laboratory.

### BA-huMAb^®^ mice

Humanized mice were usually generated by disrupting the endogenous mouse Ig genes and simultaneously introducing human heavy chain and light chain gene segments^45–48^. These mice can directly produce fully human antibodies without the need for humanization process, thus reducing the overall heterogeneity while greatly improving the speed of antibody development. Now nearly 30 fully human antibodies from transgenic mouse platforms have been approved for marketing by FDA, including Nivolumab^49^, Ipilimumab^50^, Ustekinumab^51^, and Dupilumab^52^ with ‘umab’ as their suffix. Here, BA-huMab^®^ mice were generated by introducing unrearranged human heavy chain and light chain gene segments combining with disrupting endogenous mouse heavy and light chain genes. 5'RACE and ELISA were used to ensure the expression of human immunoglobulins in these transgenic mice.

### Sequential immunization

Two mice (ID Q32 and Q35) were sequentially immunized with 40 μg recombinant RBD protein of B.1.617.1 (1st Round), B.1.617.2 (2nd Round), B.1.617.2 (3rd Round), B.1.351 (4th Round) and B.1.617.2 (final boost) in 10-day intervals. Two mice (ID Q36 and Q40) were sequentially immunized with 40 μg recombinant RBD protein of B.1.351 (1st Round), B.1.617.2 (2nd Round), B.1.617.2 (3rd Round), p.1 (4th Round) and B.1.617.2 (final boost) in 10-day intervals. Two mice (ID Q41 and Q42) were sequentially immunized with 40 μg recombinant RBD protein of B.1.617.2 (1st Round), B.1.351 (2nd Round), B.1.617.1 (3rd Round) and B.1.617.1 (final boost) in 10-day intervals. Three mice (ID Q01, Q02, and Q03) were immunized with 35 μg recombinant Spike S1 + S2 trimer Protein of B.1.617.2 variant for five rounds in 10-day intervals. Three mice (ID Q04, Q05, and Q06) were sequentially immunized with 35 μg recombinant Spike S1 + S2 trimer Protein of B.1.617.2 (1st Round), B.1.351 (2nd Round), B.1.617.2 (3rd Round), B.1.351 (4th Round) and B.1.617.2 (final boost) in 10-day intervals. Freund complete adjuvant was used in 1st round, Freund incomplete adjuvant was used in 2nd–4th rounds, and no adjuvant was used in the final booster. Spleen cells were harvested after three days of the last boost for phage libraries construction. Table 1 showed the details for the immunization process.

### Phage library construction

RNA was extracted from spleen cells of each immunized mouse by Trizol method separately. cDNA synthesis was performed using Transcriptor First Strand cDNA Synthesis Kit. The variable regions of the heavy and light chains were amplified from the cDNA by PCR separately and were integrated into the pCOMB3x vector, and then the products were electro-transfected into *Escherichia coli* TG1 for preparation of phage library. The libraries RQ32, RQ35, RQ36, RQ40, RQ41, and RQ42 were derived from mouse Q32, Q35, Q36, Q40, Q41, and Q42, respectively. The libraries SQ01, SQ02, SQ03, SQ04, SQ05, and SQ06 were derived from mouse Q01, Q02, Q03, Q04, Q05, and Q06, respectively.

### Sequential screening

Various recombinant Spike RBDs and Spike proteins were sequentially used for the panning of libraries. Plates coated with 5 μg/mL protein in CBS buffer (15 mM Na_2_CO_3_ and 35 mM NaHCO_3_, pH 9.6) or 20 μL streptavidin-magnetic beads loading 5 μg biotinylated proteins were used to capture phages with interest ScFvs. First plates were incubated with input phages at 37 °C for 2 h and beads were incubated at room temperature for 1 h. After washing 5–6 times with PBST (PBS containing 0.05%Tween-20), captured phages were eluted by Elution buffer (0.1 M HCl-Gly, pH 2.1), neutralized by 1 M Tris buffer (pH 8.0), and then used to infect 5 mL *E. coli* TG1 at 37 °C for 30 min. Helper phage VCSM13 was added for phage amplification. After overnight culture, the supernatant was collected and concentrated into a phage library for next round panning. After three rounds panning, TG1 cells infected eluted phages were grown on plates. ScFvs were expressed and their binding and blocking activity was tested by ELISA. Positive hits were obtained and sequenced.

### Human monoclonal antibodies and bispecific antibody

Recombinant antibody heavy chain variable region and light chain variable region were amplified (2× Phanta Max Master Mix, Vazyme, P515-01) using the positive clones screened from the library as the template. Heavy chain variable region was fused (ClonExpress II One Step Cloning Kit, Vazyme, C112-01) into the linearized pcDNA3.4 vectors with human IgG1 constant region. Light chain variable region was fused (ClonExpress II One Step Cloning Kit, Vazyme, C112-01) into the linearized pcDNA3.4 vectors with human κ constant region. For engineering BA7208-7125, ScFv of 7125 with a linker (GGSGGGSGGGSGGGSGGGSG) between VL and VH was fused to Hinge-CH2-CH3 of IgG1 via an AA linker, and the S354C and T366W mutations were introduced into the heavy chain CH3 region to generate the hole. Meanwhile, the mutations Y349C, T366S, L368A, and Y407V mutations were introduced into the CH3 region of the heavy chain of BA7208. The sequences of VIR-7381, REGN-1033, and REGN10987 were all derived from IMGT Database. The recombinant plasmids were prepared for antibody expression. Antibodies were expressed with Expi-CHO Expression system (Gibco) for 10–12 d and the supernatants were harvested and purified by protein A resin (BestChrom, AA0272) and Chromdex 200 PG resin (BestChrom, AG0083) sequentially. BA7208/7125 was further purified by SP Sepharose High Performance resin (Cytiva, 17-0729-10).

### ELISA-based receptor-binding inhibition assay

High-binding ELISA plates were coated with 0.125 μg/mL recombinant SARS-CoV-2 RBD of original WT strain or B.1.351 or B.1.617.2 or B.1.1.529 variant at 4 °C overnight, and then blocked with 3% skim milk powder in PBST at 37 °C for 1 h. ScFv or serially diluted antibody was mixed with biotinylated human ACE2 (final concentration 0.04 μg/mL) and then the mixture was incubated with coated RBD in the plates for 1 h at 37 °C. After washing two times, the retained biotinylated ACE2 binding to coated RBD was detected by HRP-conjugated Streptomycin. Inhibition rate % = (OD_450_ of no antibody−OD_450_)/OD_450_ of no antibody*100%. Experiments were performed in triplicate for Supplementary Fig. S3, value = mean ± SD.

### Pseudovirus neutralization assay

VSV pseudotyped with SARS-CoV S protein provided by Beijing SanYao Science & Technology Development Co. were produced and titrated as described previously^53^. 50 μL SARS-CoV-2 pseudovirus (2.0 × 10^4^ TCID_50_/mL) were incubated with 100 μL 0.19 μg/mL or 3-fold serially diluted antibodies at 37 °C for 1 h, and then 100 μL cell suspensions of human Huh7 cells (4 × 10^5^ cells/mL) were added to the mixtures. The mixture of 100 μL DMEM medium and 50 μL pseudovirus was used as negative control and 150 μL DMEM was used as blank control. After 20–28 h incubation at 37 °C, neutralizations potencies of mAbs were evaluated in a luciferase assay (PerkinElmer). 150 μL supernatant was abandoned and 100 μL britelite plus was added as a substrate (PerkinElmer). Luminous value was detected by Microplate reader (TECAN, Infinite M200 Pro) and inhibitory rate was calculated by (1−(mean RLU of sample–mean RLU of blank control)/(mean RLU of negative control−mean RLU of blank control)*100%). The half maximal inhibitory concentrations (IC_50_) were determined using 4-parameter logistic regression (GraphPad Prism). Experiments were performed in duplicate, value = mean ± SD.

### Affinity to SARS-CoV-2 Spike-RBD from SARS-CoV-2 variants

The binding kinetics was assessed by SPR assay using the BIAcore 8 K system. The equilibrium constant (*K*_d_) measurements were performed at room temperature with ProA chip. HBS-EP + buffer (150 mM NaCl, 10 mM HEPES, 3 mM EDTA, and 0.05% (v/v) surfactant P20 pH 7.4) was used as running buffer. The blank channel of the chip served as the negative control. Antibody was captured on the chip at 400–500 response units. Serial dilutions of SARS-CoV-2 RBD proteins (from 50 nM to 3.125 nM with twofold dilution, for BA7208-7125 from 25 nM to 1.5625 nM with twofold dilution) in running buffer were applied to flow over the chip surface. After dissociation for 450 s, the chip was regenerated for 30 s with 10 mM pH 1.5 Glycine after each cycle. The affinity was calculated using a 1:1 (Langmuir) binding fit model with BIA evaluation software. Experiments were performed in duplicate, value = mean ± SD.

### BLI based competitive binding assay

Competitive binding of the antibodies was performed on a ForteBio Octet Red96 system (Pall Forte BioCorporation, Menlo Park, CA) using in-tandem format binning assay. Biotinylated RBD protein of Delta variant (Sino Biological, Cat. 40592-V08H90) was loaded onto SA sensors (Fortebio, Cat. 18-5019). The sensors were then exposed to the first antibody with 30 µg/mL or PBST for 100 s, then to the second antibody at 30 µg/mL for 100 s. Data were processed using ForteBio’s Data Analysis Software 9.0.

### Neutralizing assay by FRNT

FRNT was performed in a certified Biosafety level-3 lab as previously described^54^. In detail 50 μL antibodies were serially diluted, mixed with 50 μL of SARS-CoV-2 (180 focus forming unit, FFU) in 96-well microwell plates, and incubated for 1 h at 37 °C. Mixtures were then transferred to 96-well plates seeded with Vero E6 cells (ATCC, Manassas, VA) for 1 h at 37 °C to allow virus entry. Inoculums were then removed before adding the overlay media (100 μL MEM containing 1.2% Carboxymethylcellulose, CMC). The plates were then incubated at 37 °C for 24 h. Overlays were removed and cells were fixed with 4% paraformaldehyde solution for 30 min. Cells were permeabilized with 0.2% Triton X-100 and incubated with cross-reactive rabbit anti-SARS-CoV-N IgG (Sino Biological, Cat 40143-R001) for 1 h at room temperature before adding HRP-conjugated goat anti-rabbit IgG(H + L) antibody (1:4000 dilution) (Jackson ImmunoResearch, West Grove, PA). Cells were further incubated at room temperature. The reactions were developed with KPL TrueBlue Peroxidase substrates (Seracare Life Sciences Inc, Milford, MA). The numbers of SARS-CoV-2 foci were calculated using an EliSpot reader (Cellular Technology Ltd, Shaker Heights, OH).

### Cryo-EM sample preparation and data acquisition

For the complexes of Delta-Spike/BA7208-Fab/BA7125-Fab, Delta-Spike/BA7054-Fab/BA7125-Fab, and Omicron-Spike/BA7208-Fab, C-flat R 2/1 holey carbon grids were first glow discharged for 15 s using a Pelco easiGlow glow discharge unit and 3.5 μL sample was applied to the surface of the grid at a temperature of 6 °C and a humidity level of 90%. Grids were then blotted for 2 s before being plunge-frozen in liquid ethane using Vitrobot Mark IV. Grids were imaged using a 300 kV Titan Krios electron microscope (Thermo Fisher Scientific) equipped with a GIF-Quantum energy filter (Gatan), which was used with a slit width of 20 eV. Automatic data collection was performed using EPU software. Images were recorded with a Gatan K3 direct electron detector operating in super-resolution counting mode at pixel size of 0.66 Å for the Delta-S/7208/7125 dataset, 1.072 Å for the Delta-S/7054/7125 dataset, and 0.856 Å for the Omicron-S/7208 dataset. The exposure was performed with a dose rate of 15 e-/pixel/s and an accumulative dose of ~50 e-/Å^2^ for each image which was fractionated into 36 movie-frames. The final defocus ranges of the datasets were ~−(1.5–2.5) μm for the Delta-S/7208/7125 dataset, –(1.0–2.0) μm for both the Delta-S/7054/7125 and 0.856 Å for the Omicron-S/7208 datasets.

### Image processing and 3D reconstruction

For all three datasets, the dose-fractionated image stacks were subjected to beam-induced motion correction using MotionCor2. Initial contrast transfer function (CTF) values for each micrograph were calculated with CTFFIND4. Micrographs with an estimated resolution limit worse than 4 Å were discarded in the initial screening. A set of ~150,000 particles were blob-picked and subjected to 2D classification to generate templates for auto-picking against the entire dataset. The subsequent image processing and reconstruction were performed using cryoSPARC. 1,148,586/1,659,975/633,788 particles were picked from the micrographs. Then the picked particles were extracted and subjected to three rounds of reference-free 2D classification in cryoSPARC, which yielded 309,383/335,175/439,731 particle projections. This subset was subjected to one round of hetero refinement and the complex subsets were used to obtain density maps with resolution of 3.08/3.21/2.62 Å for the three datasets. Local refinement focused on the RBD/BA7208-Fab/BA7125-Fab and RBD/BA7054-Fab/BA7125-Fab with mask could reconstitute the structure at a 2.98/3.18 Å resolution. Local resolution estimate was performed with cryoSPARC.

### Model building

The structure of the spike protein (PDB: 6XCN), was docked into the cryo-EM density maps using CHIMERA. The models were manually corrected for local fit in COOT and the sequence register was updated based on alignment. The models were refined against corresponding maps in real space using PHENIX, in which the secondary structural restraints and Ramachandran restrains were applied. The stereochemical quality of each model was assessed using MolProbity. Statistics for model refinement and validation are shown in Supplementary Tables S1 and S4.

### Animal experiments

The in vivo prophylactic and therapeutic potency of BA7208 and bispecific antibody BA7208/7125 against SARS-CoV-2 was evaluated in hACE2-transgenic (K18) and WT BALB/c mice (wt) as previously described^37^. In all, 6–8 weeks old mice were intraperitoneally injected with 200 μg (10 mg/kg) of BA7208 or BA7208/7125 per mouse 24 h before or 4 h after the challenge with 1 × 10^5^ FFU SARS-CoV-2 (Omicron BA.1 or BA.2). Mice injected with phosphate-buffered saline (PBS) were challenged with the same dose of SARS-CoV-2 as controls. To investigate the presence of SARS-CoV-2 in the lungs, lungs were harvested for viral titers 1 d later by using FFA. Meanwhile, we used an aerosol inhalation apparatus to administer the antibody for evaluation of its protection in vivo. After administration of antibody via aerosol inhalation (3 mg/kg) or intranasal (1 mg/kg), we collected the lung 1 d after infection for viral titers.

### Pharmacokinetics studies

A single intravenous injection of antibodies (BA7208, BA7125, and BA7208/7125) was conducted in Balb/c mice (*n* = 9, 3/group, age 5–7 weeks, body weight 25 ± 3 g) at 10 mg/kg. Blood samples were collected at predose and 5 min, 30 min, 1 h, 4 h, 8 h, 24 h, 72 h, 120 h, 168 h, 240 h, 336 h postdose from the mice via orbit vein bleeding. After placed at room temperature for 30 min, the blood samples were centrifuged at 13500 rpm for 10 min at 4 °C, and supernatants were analyzed by ELISA. High-binding ELISA plates were coated with 1 μg/mL SARS-CoV-2 RBD protein of B.1.617.2 variant at 4 °C overnight, and then blocked with 1% BSA in PBS at 37 °C for 1 h, followed by washing five times with PBST. Serially diluted antibodies (for standard curve) and 4-fold or 20-fold dilutions of serum samples were added to the plates and incubated at 37 °C for 1 h, followed by washing five times with PBST. Goat Anti-Human IgG-HRP (Southern Biotech, 2049-05) was used as a secondary antibody. Concentration of antibodies in serum was calculated according to the standard curve. The main PK kinetic parameters were calculated using Phoenix WinNonlin. Values are shown as mean ± SD.

### Statistics and reproducibility

All statistical analyses were performed in GraphPad Prism 8 software, except for affinity data which was performed in Excel. Details on the statistical tests applied are provided in figure legends. The data are reported as bar graphs displaying individual value and mean ± SD, as indicated in the figure legends. No experiments were excluded from the analyses.

## Supplementary information

Supplementary Figures and Tables

## Data Availability

The cryo-EM maps of the SARS-CoV-2 Delta Spike protein in complex with BA7208-Fab and BA7125-Fab, the SARS-CoV-2 Delta RBD in complex with BA7208-Fab and BA7125-Fab (local refinement), the SARS-CoV-2 Omicron Spike protein in complex with BA7208-Fab have been deposited in the Electron Microscopy Data Bank under accession codes EMD-33151, EMD-31140, and EMD-33142, respectively. The atomic coordinates of above complexes have been deposited in the Protein Data Bank under the accession code 7XDL, 7XDA, and 7XDB, respectively. The sequences of mAbs have been deposited in GenBank with the accession codes ON092631-ON092638 for BA7208 heavy chain, BA7208 light chain, BA7125 heavy chain, BA7125 light chain, BA7054 heavy chain, BA7054 light chain, BA7134 heavy chain and BA7134 light chain respectively. The data used to support the findings of this study are included within the article.
