# Supplementary material for: Biparatopic antibody BA7208/7125 effectively neutralizes SARS-CoV-2 variants including Omicron BA.1-BA.5
Source: Cell Discov. 2023 Jan 7;9:3. doi: 10.1038/s41421-022-00509-9 (PMC9822811; doi:10.1038/s41421-022-00509-9)
Supplement: Supplementary file 1 — Supplementary Figures and Tables [file 41421_2022_509_MOESM1_ESM.pdf]

Supplementary Materials for

Biparatopic antibody BA7208/7125 effectively neutralizes SARS-CoV-2 variants including Omicron BA.1-BA.5

Yanqun Wang[1,2,13], An Yan[3,13], Deyong Song[4,13], Chuangchuang Dong[4], Muding Rao[4], Yuanzhu Gao[3], Ruxi Qi[3], Xiaomin Ma[3], Qiaoping Wang[4], Hongguang Xu[4], Hong Liu[4], Jing Han[4], Maoqin Duan[5,] Shuo Liu[5], Xiaoping Yu[4], Mengqi Zong[4], Jianxia Feng[4], Jie Jiao[4], Huimin Zhang[4], Min Li[4], Beibei Yu[4], Yanxia Wang[6], Fanhao Meng[6], Xiaodan Ni[6], Ying Li[4], Zhenduo Shen[4], Baiping Sun[4], Xin Shao[4], Haifeng Zhao[4], Yanyan Zhao[4], Rui Li[4], Yanan Zhang[4], Guangying Du[7], Jun Lu[4], Chunna You[7], Hua Jiang[4], Lu Zhang[8], Lan Wang[5*], Changlin Dou[4*], Zheng Liu[3*], Jincun Zhao[1,9,10,11,12*]

[13]Co-first author

Corresponding:

*Correspondence author: Jincun Zhao (zhaojincun@gird.cn), Zheng Liu (liuz3@sustech.edu.cn), Changlin Dou (douchanglin@boan-bio.com), Lan Wang (wanglan@nifdc.org.cn)

Lead contact：Dr. Jincun Zhao

This PDF file includes:

Methods for Binding free energy calculation

Methods for the structure study of the Omicron S-BA7208 complex

References for the additional methods

Figure S1 to S10

Table S1 to S9

Methods

Binding free energy calculation

To calibrate the binding affinities of the S protein with antibodies and predict the binding ability of the BA7054-7125-RBD complexes which lacked the experimental data, we used the Flex ddG protocol[1] implemented within Rosetta to calculate the changes in binding free energies (interface $\Delta\Delta G$) upon mutation. All Spikes were truncated to include only the RBD domain in the S protein RBD-antibody complexes. The data from the Delta Spike RBD (monomer)-antibody were set as the references, with which the values of all other variants were compared. The $\Delta\Delta G$ calculation of each system was repeated 50 times and the average values with two different scoring types were reported.

Cryo-EM specimen preparation and data acquisition for the Omicron S-BA7208 complex

90 μL purified Omicron S protein at the concentration of 1.37 mg/mL was incubated with 10.1 μL BA7208 Fab at the concentration of 3.99 mg/mL at a 1:3 molar ratio on ice for 40 min for the next step of size exclusion chromatography (SEC). After centrifugation (16200 g, 4°C for 5 min), 100 μL supernatant was injected to GE micro Akta using Superose 6 column. The peak fraction was applied for cryo-EM grid preparation.

An aliquot of 4 μL protein sample of Omicron S-BA7208 complex was applied onto a glow-discharged 300 mesh grid (Quantifoil Au R1.2/1.3) supported with a thin layer of GO (Graphene Oxide), blotted with filter paper for 3.0 s and plunge-frozen in liquid ethane using a Thermo Fisher Vitrobot Mark IV. Cryo-EM micrographs were collected on a 300kV Thermo Fisher Titan Krios G3i electron microscope equipped with a K3 direct detection camera and a BioQuantum image filter (GIF: a slit width of 20eV). The micrographs were collected at a calibrated magnification of x81,000, yielding a pixel size of 0.856 Å at a super-resolution mode. In total, 2,554 micrographs were collected at an accumulated electron dose of $50e^{-}Å^{-2}\ s^{-1}$ on each

micrograph that was fractionated into a stack of 32 frames with a defocus range of -1.0 μm to −2.0 μm.

EM data processing

Beam-induced motion correction was performed on the stack of frames using MotionCorr2[2]. The contrast transfer function (CTF) parameters were determined by CTFFIND4[3]. A total 2,554 good micrographs were selected for further data processing using Relion 3.1 program[4]. Particles were auto-picked by the Auto-picking program in Relion, followed by two rounds of reference-free 2D classifications. Next, 633,788 particles were selected from good 2D classes and were subjected to two rounds of 3D classification using a reconstruction of the Omicron S-BA7208 complex as a starting model. Two converged 3D classes with a feature contains one Omicron S-BA7208, one Omicron S were selected for a final round of 3D refinement. In the two 3D classes, 495,537 particles from a 3D class showing the highest resolution feature with an additional density were selected for a round of 3D refinement, yielding a final reconstruction at a global resolution of 2.62 Å based on the gold-standard Fourier shell correlation criterion at FSC=0.143. The local resolution was then calculated on the final density map.

Model building and refinement

The model of Omicron S-BA7208 complex was built by fitting the model of structure of Omicron S-BA7208 (predicted by AlphaFold2) into the density map using UCSF Chimera[5,6], followed by a manual model building of Omicron S-BA7208 complex molecules in COOT[7] and a real space refinement in PHENIX[8]. The model statistics were listed in Supplementary information, Table S7.

References

1 Barlow, K. A. *et al.* Flex ddG: Rosetta Ensemble-Based Estimation of

Changes in Protein-Protein Binding Affinity upon Mutation. **122**, 5389-5399, doi:10.1021/acs.jpcb.7b11367 (2018).

2 Zheng, S. Q. *et al.* MotionCor2: anisotropic correction of beam-induced motion for improved cryo-electron microscopy. *Nat Methods* **14**, 331-332, doi:10.1038/nmeth.4193 (2017).

3 Rohou, A. & Grigorieff, N. CTFFIND4: Fast and accurate defocus estimation from electron micrographs. *J Struct Biol* **192**, 216-221, doi:10.1016/j.jsb.2015.08.008 (2015).

4 Zivanov, J. *et al.* New tools for automated high-resolution cryo-EM structure determination in RELION-3. *Elife* **7**, doi:10.7554/eLife.42166 (2018).

5 Pettersen, E. F. *et al.* UCSF Chimera--a visualization system for exploratory research and analysis. *J Comput Chem* **25**, 1605-1612, doi:10.1002/jcc.20084 (2004).

6 Bryant, P., Pozzati, G. & Elofsson, A. Improved prediction of protein-protein interactions using AlphaFold2. *Nat Commun* **13**, 1265, doi:10.1038/s41467-022-28865-w (2022).

7 Bond, P. S., Wilson, K. S. & Cowtan, K. D. Predicting protein model correctness in Coot using machine learning. *Acta Crystallogr D Struct Biol* **76**, 713-723, doi:10.1107/S2059798320009080 (2020).

8 Adams, P. D. *et al.* PHENIX: a comprehensive Python-based system for macromolecular structure solution. *Acta Crystallogr D Biol Crystallogr* **66**, 213-221, doi:10.1107/S0907444909052925 (2010).

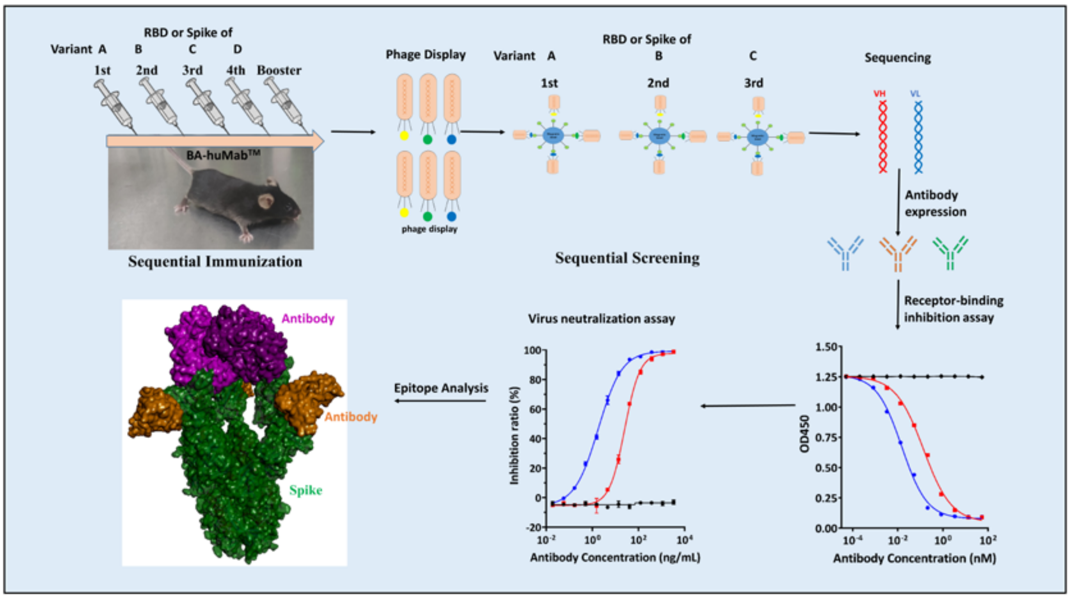

Fig. S1 Graphical Abstract for antibody identification and characterization mAbs against SARS-CoV-2 variants were produced through sequential immunization and followed sequential screening from human antibody transgenic mice. ScFvs of potential clones were sequenced and then converted into IgG format for expression. Antibodies showing RBD/ACE2 receptor blocking activity were obtained for *in vitro* neutralization activity evaluation. Structure analysis was performed for the broadly potent neutralizing mAbs.

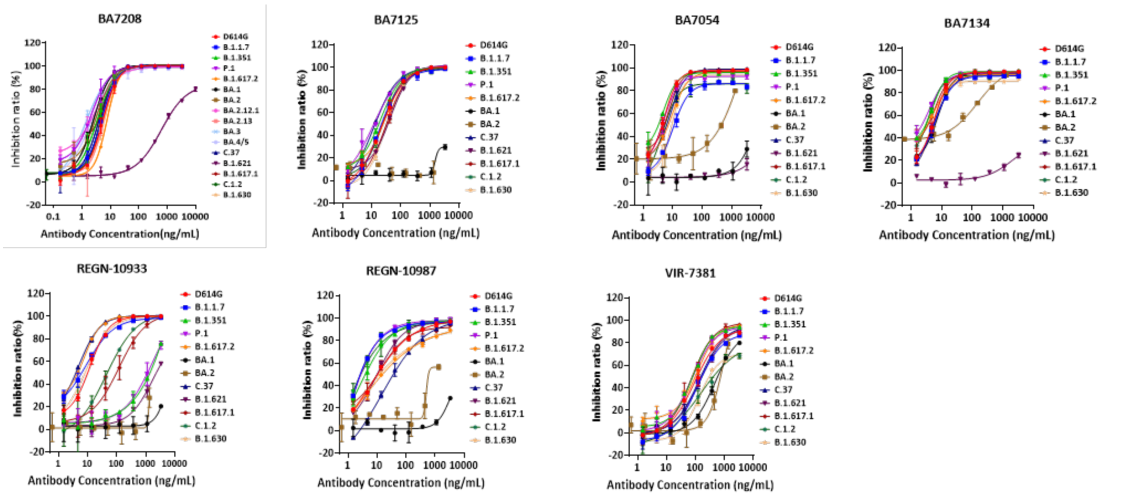

Fig. S2 Neutralization curves of anti-SARS-CoV-2 antibodies against diverse SARS-CoV-2 variants. Neutralization curves of the indicated antibodies against the pseudoviruses decorated with the corresponding SARS-CoV S proteins are shown. Data was collected from two biological replicates and represented as Mean±SD.

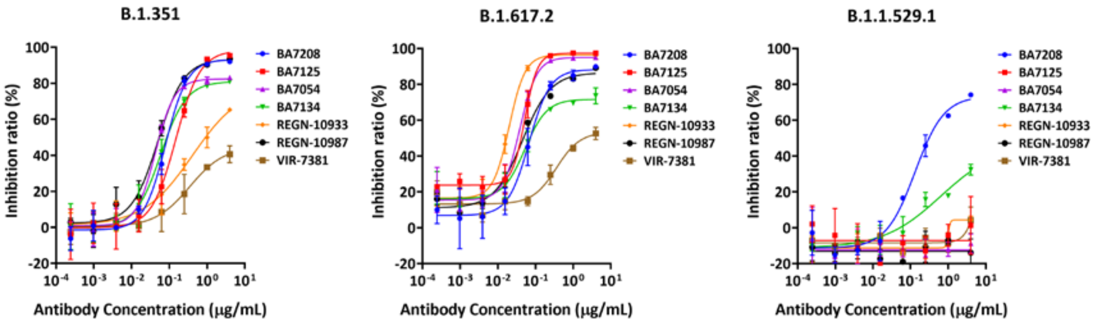

Fig. S3 Blocking activity of anti-SARS-CoV-2 antibodies for RBD-ACE2 binding

Blocking activity of four candidate antibodies and three approved antibodies against RBDs of Beta (B.1.351), Delta (B.1.617.2) and BA.1 (B.1.1.529.1) binding to hACE2 was analyzed in ELISA-based receptor-binding inhibition assay. Experiments were performed in triplicate, value = Mean ± SD.

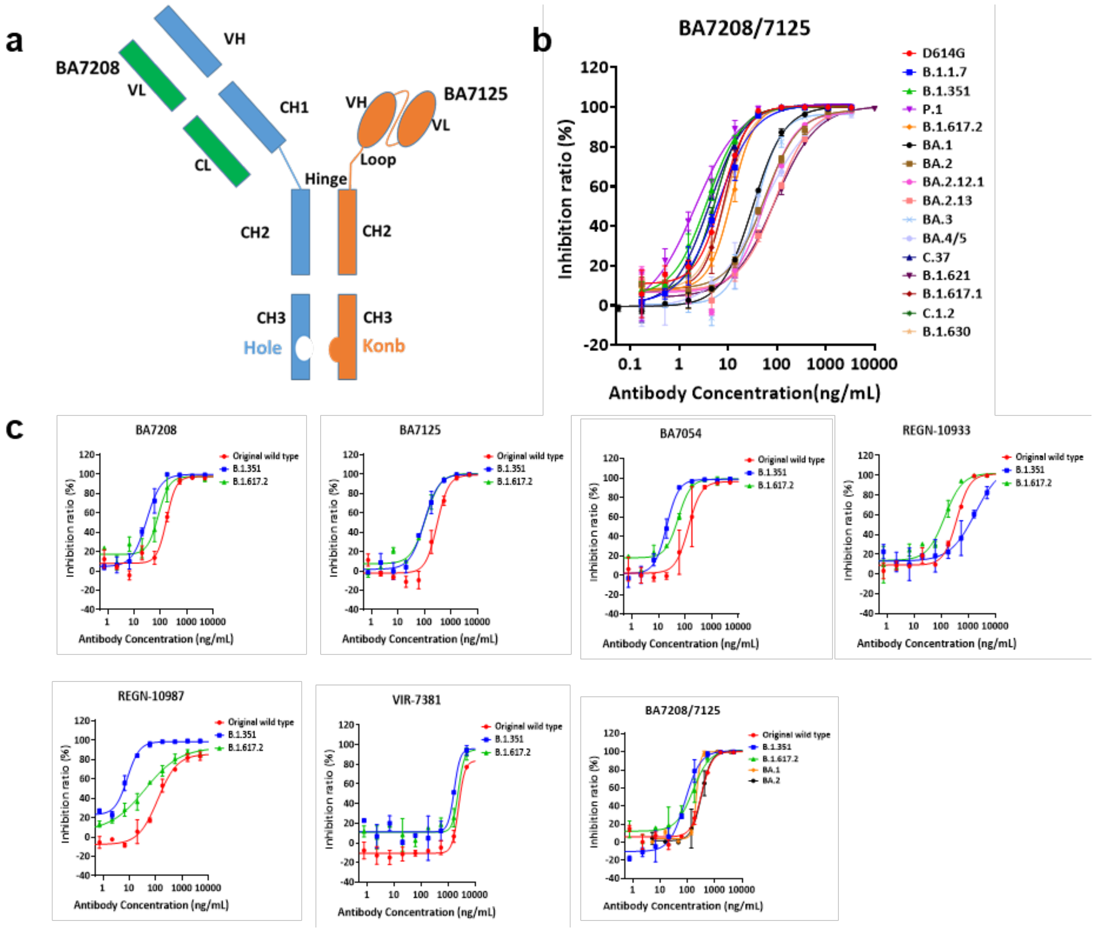

Fig. S4 Comparison of neutralizing activity and affinity between the bispecific BA7208/7125 and its parental antibodies. a Schematic diagram of the bispecific BA7208/7125. The Knob-into-Hole design was used in the Fc region. **b** Neutralization curves of the indicated antibodies against the pseudoviruses decorated with the corresponding SARS-CoV S proteins are shown. Data was collected from two biological replicates and represented as Mean±SD. **c** Neutralization curves against the authentic SARS-CoV-2 variants are shown for each of the indicated antibodies. Two biological replicates were performed.

a

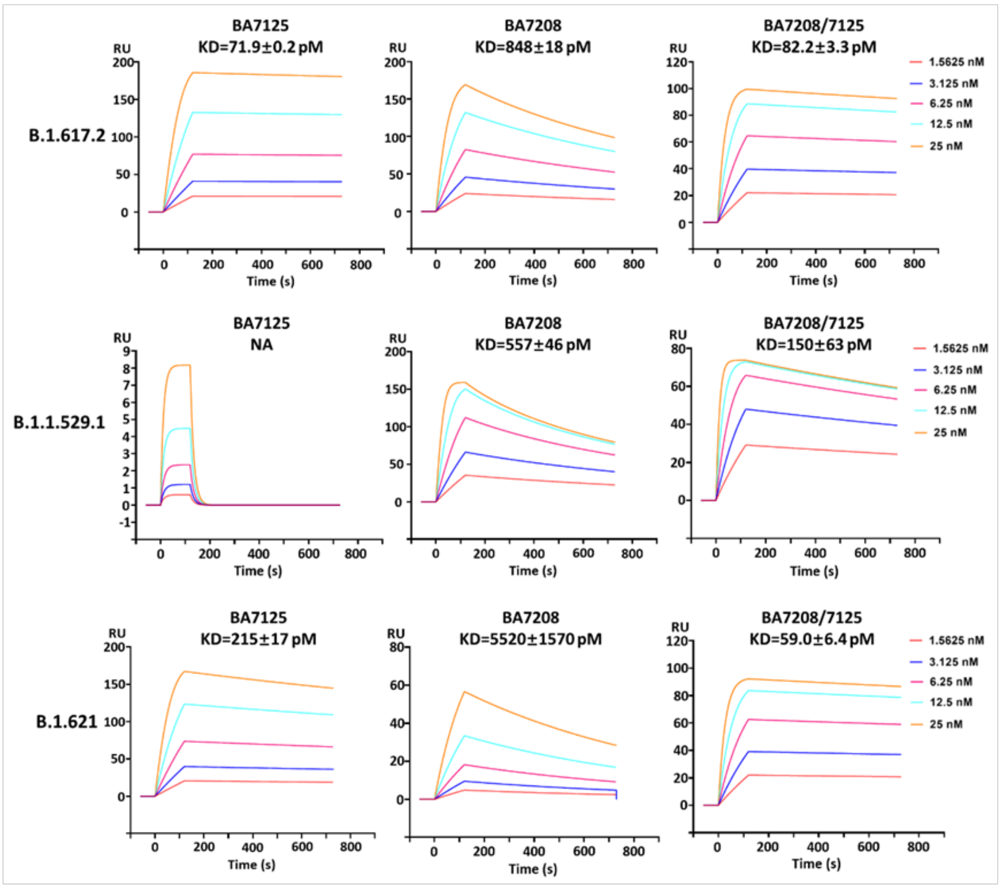

b

Antibody ID	KD(pM) by Biacore		
	Delta B.1.617.2	Mu B.1.621	Omicron BA.1 B.1.1.529
BA7125	71.9±0.2	215±17	NA
BA7208	848±18	5520±1570	557±46
BA7208/7125	82.2±3.3	59.0±6.4	150±63

Fig. S5 The binding kinetics of the bispecific BA7208/7125 and its parental antibodies to RBD of B.1.617.2, B.1.1.529.1 and B.1.621 variants. a Antibodies were immobilized onto protein A biosensors and the RBD-His was in solutions. The binding sensorgrams for indicated antibodies were shown with serially diluted RBD proteins in different colors. KD data was collected from two biological replicates and represented as Mean±SD. **b** KD values of the indicated antibodies to RBD of Omicron BA.1 (B.1.1.529.1), Delta and Mu variants examined by SPR. NA: little to no binding.

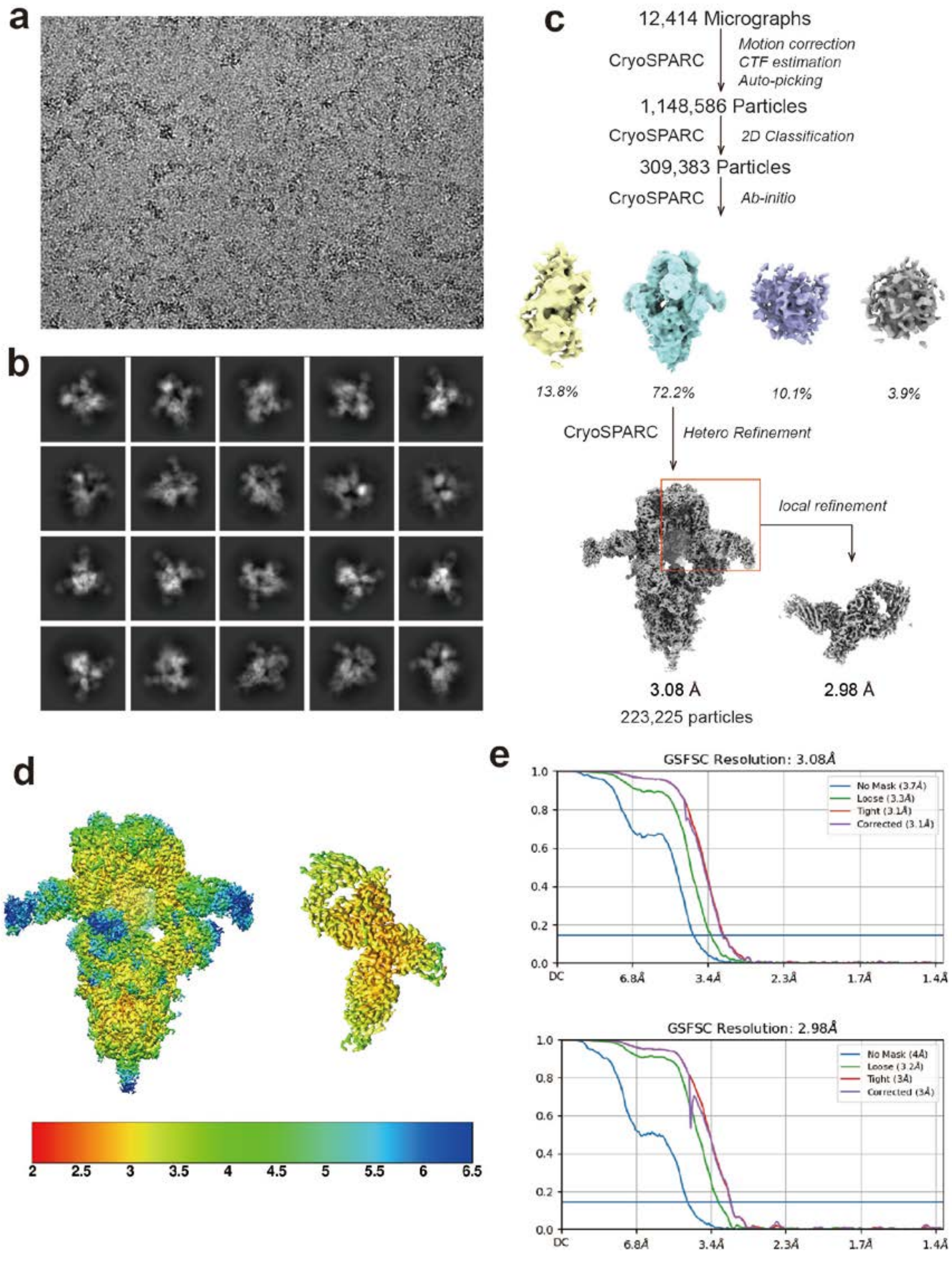

Fig. S6 Cryo-EM data processing of Delta Spike/BA7208-Fab/BA7125-Fab. a Representative cryo-EM micrograph of Delta-Spike/BA7208-Fab/BA7125-Fab. **b** 2D class average images of Delta-Spike/BA7208-Fab/BA7125-Fab. **c** Brief workflow of cryo-EM image processing and reconstruction. **d** Cryo-EM map of Delta-Spike/BA7208-Fab/BA7125-Fab, and Delta-RBD/BA7208-Fab/BA7125-Fab colored by local resolution (Å). **e** The FSC curves for the reconstructions.

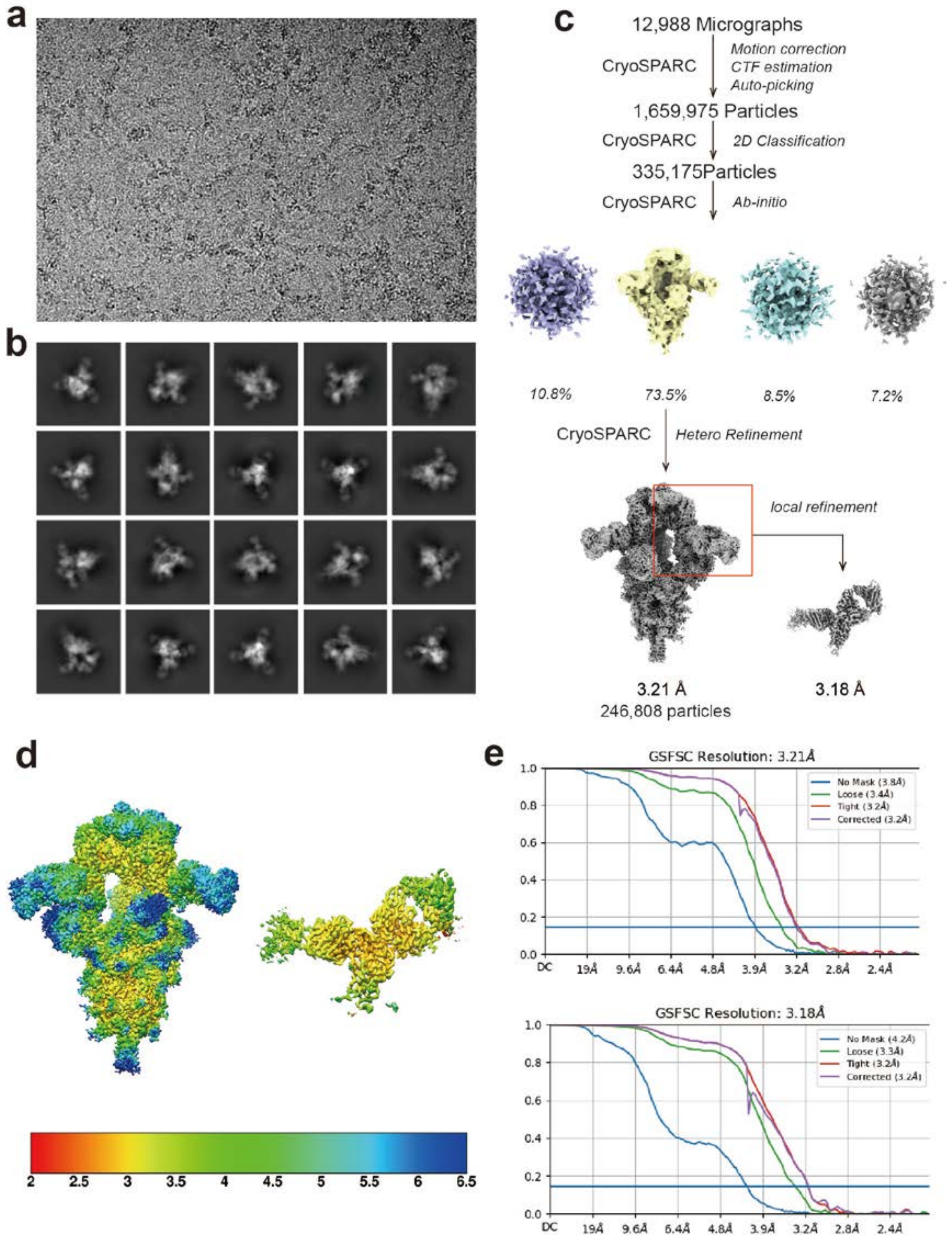

Fig. S7 Cryo-EM data processing of Delta-Spike/BA7054-Fab/BA7125-Fab. a Representative cryo-EM micrograph of the Delta-Spike/BA7208-Fab/BA7125-Fab. **b** 2D class average images of Delta-Spike/BA7208-Fab/BA7125-Fab. **c** Brief workflow of cryo-EM image processing and reconstruction. **d** Cryo-EM map of Delta-Spike/BA7208-Fab/BA7125-Fab, and Delta-RBD/BA7208-Fab/BA7125-Fabcolored by local resolution (Å). **e** The FSC curves for the reconstructions.

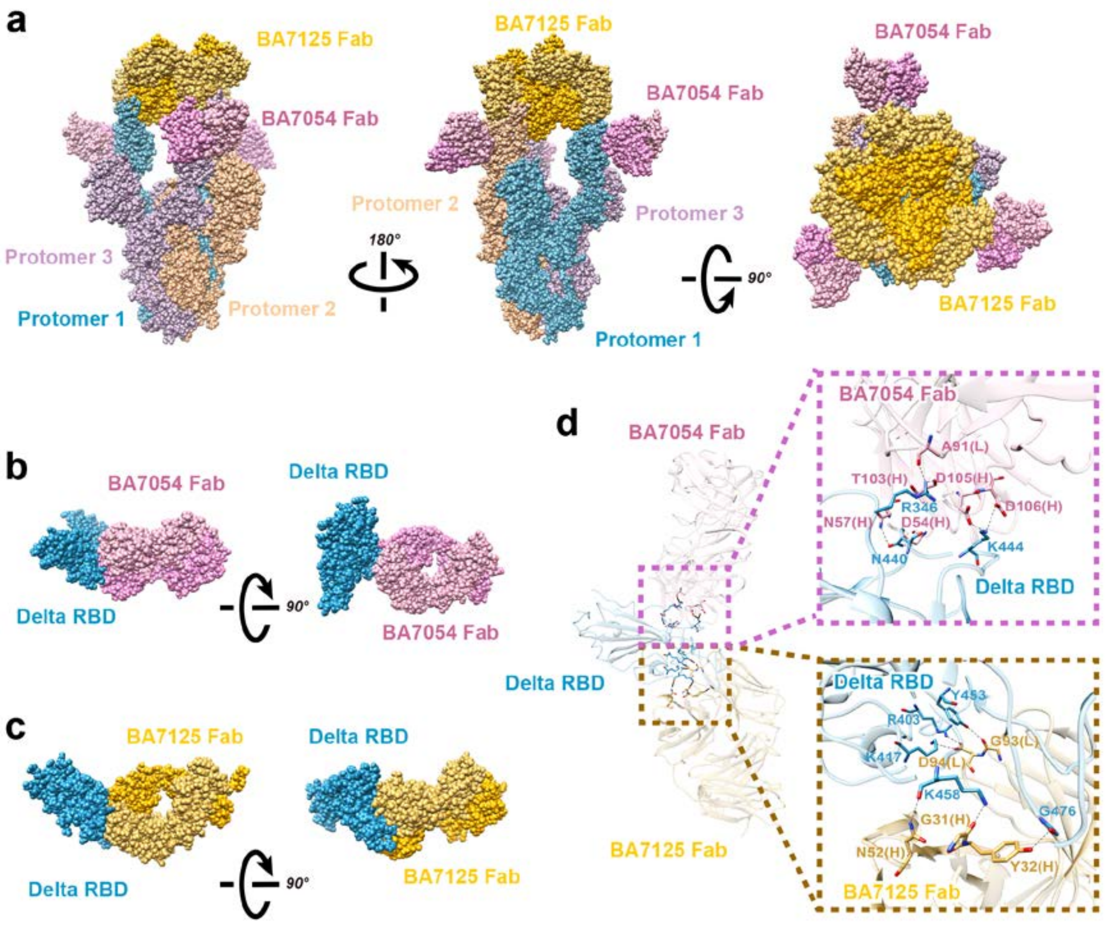

Fig. S8 Cryo-electron microscopy of Delta Spike Trimer with BA7054-Fab/BA7125-Fab. a The complex of three BA7054-Fabs (pink) and three BA7125-Fab (yellow) with Delta Spike Trimer (dodger blue, plum, and rosy brown). **b** The complex of BA7054-Fab (light pink, Fab light chain; dark pink, Fab heavy chain) with Delta Spike RBD (dodger blue). **c** The complex of BA7125-Fab (light yellow, Fab light chain; dark yellow, Fab heavy chain) with Delta Spike RBD (dodger blue). **d** Zoomed-in views of Fabs binding sites on Delta RBD, side chain of residues that form the hydrogen bonds and salt bridges are displayed.

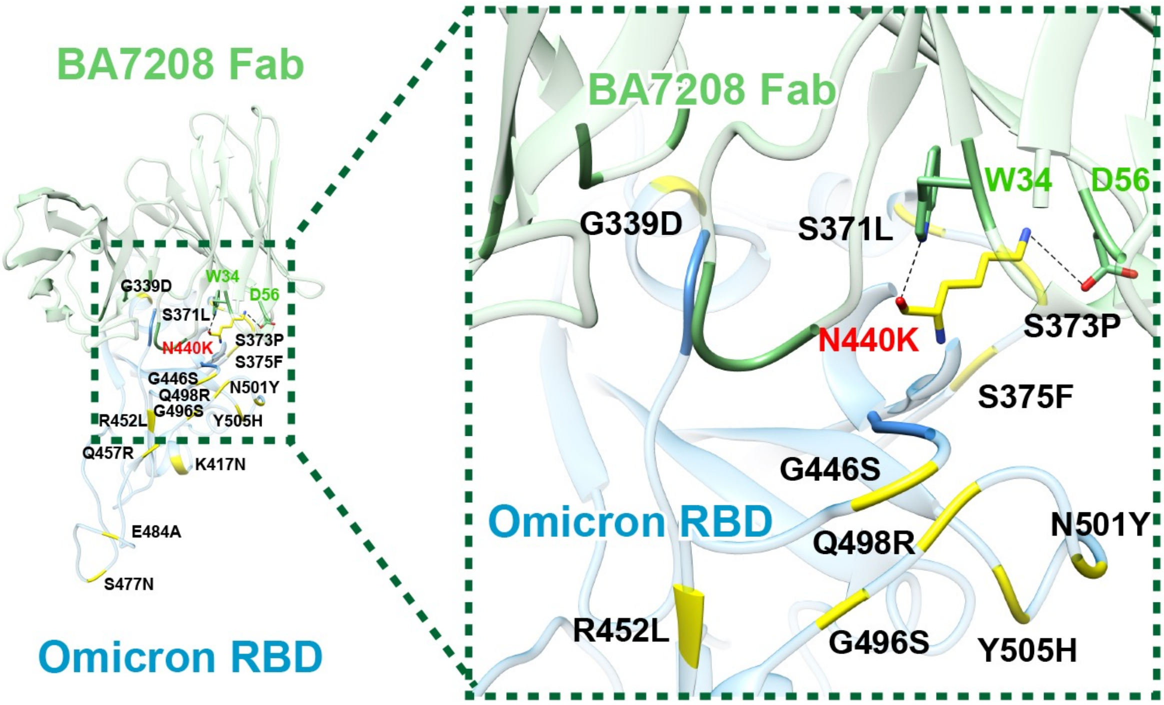

Fig. S9 Mutational residues in Omicron RBD are labeled, only N440K (red) interacts directly with residues W34 and D56 (green) in BA7208 Fab heavy chain.

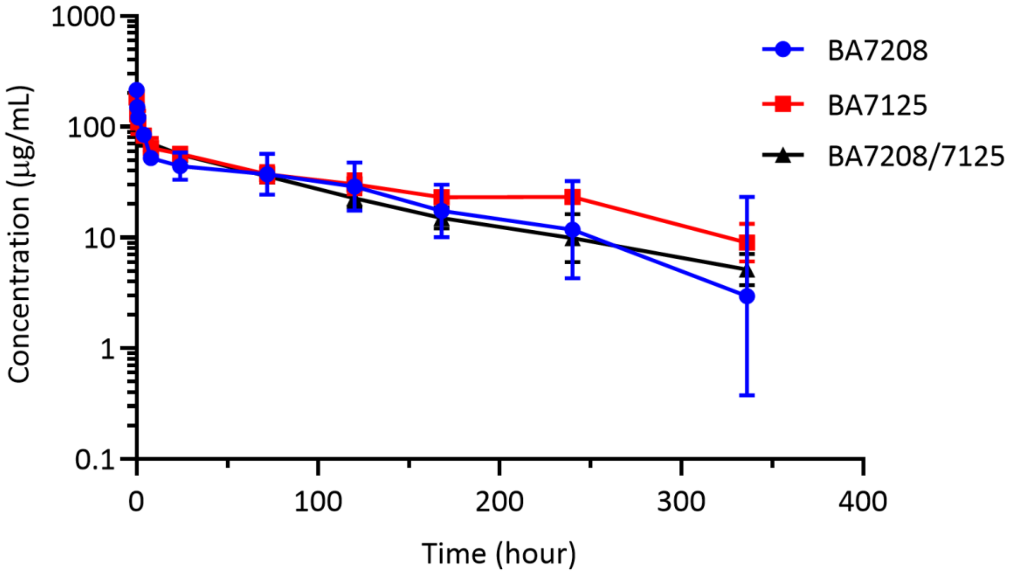

Fig. S10 Log-linear serum concentration versus time profiles of BA7208, BA7125 and BA7208/7125 in mice. Mice (BALB/c) were administered intravenously at a dose of 10 mg/kg with BA7208, BA7125 and BA7208/7125. Antibody concentrations in serum were determined in Elisa with RBD protein of Delta variant as the capture reagent. Results are shown as Mean ± SD (N = 3). The main PK kinetic parameters were calculated using Phoenix WinNonlin.

Table S1. Binding free energy calculation. The systems with relatively higher binding free energies than the Delta system are highlighted in blue, and those with lower ones are highlighted in orange. The BA7208 shows good binding ability for all kinds of Spike RBD. While the BA7054 and BA7125 show weaker bindings for Omicron Spike RBD, especially in the BA7125-RBD systems. These results are consistent with the experimental data we observed. Of note is the BA7054-7125-RBD systems, where the binding ability for Omicron is not as good as other variants.

BA7054-RBD

ddG	Type0	Type1
Delta	0	0
Alpha	0.00±0.01	-0.06±0.05
Beta	-0.04±0.04	-0.03±0.03
Gamma	-0.02±0.04	-0.02±0.05
Omicron	1.90±0.20	2.02±0.81

BA7125-RBD:

ddG	Type0	Type1
Delta	0	0
Alpha	0.25±0.09	0.17±0.08
Beta	0.57±0.19	0.60±0.25
Gamma	0.92±0.22	0.83±0.37
Omicron	6.50±1.62	6.80±1.85

BA7208-RBD:

ddG	Type0	Type1
Delta	0	0
Alpha	0.04±0.01	0.04±0.02
Beta	0.31±0.06	0.32±0.23
Gamma	0.32±0.03	0.31±0.04
Omicron	0.24±0.14	0.30±0.31

BA7054-7125-RBD:

ddG	Type0	Type1
Delta	0	0
Alpha	0.02±0.10	0.00±0.13
Beta	-0.12±0.31	-0.07±0.35
Gamma	-0.44±0.29	-0.35±0.40
Omicron	3.86±4.95	0.90±5.19

BA7208-7125-RBD:

ddG	Type0	Type1
Delta	0	0
Alpha	0.68±1.88	0.84±1.33
Beta	-1.73±0.68	-1.72±0.94
Gamma	-1.53±0.77	-1.43±1.02
Omicron	-8.06±3.14	-6.67±3.94

Table S2. Cryo-EM data collection, refinement and validation statistics for the structure of Delta S with BA7125-Fab and BA7208-Fab.

	Delta S-BA7208/BA7125 Consensus map
**Data collection and image processing**	
Microscope	FEI Titan Krios
Camera	Gatan K3
Magnification	74,880
Voltage(kV)	300
Total dose (e–/Å2)	50
Defocus range (μm)	-1.5 to -2.5
Pixel size (Å/pixel)	0.66
Frames/movie	36
Movies (total)	12,414
Initial particles images	1,148,586
Final particles images	309,383
Symmetry imposed	C1
**Map resolution (Å)**	3.08
FSC threshold	0.143
Map resolution range (Å)	2.0-6.5
**Refinement**	
Initial model used (PDB code)	6XCN
**Model composition**	
Non-hydrogen atoms	39,008
Protein residues	4,985
Ligand	30
**R.m.s. deviations**	
Bond lengths (Å)	0.005
Bond angles (°)	0.701
**Validation**	
MolProbity score	2.15
Clashscore	10.85
Poor rotamers (%)	0.35
**Ramachandran plot**	
Favored (%)	92.83
Allowed (%)	7.17
Disallowed (%)	0.00

Table S3. Protein-protein interaction between Delta RBD and BA7208 Fab.

	Residue in Delta RBD	Residue in BA7208 Fab	Distance (Å)
Hydrogen bond	Thr(T)-345	Phe(F)-94	3.3
	Arg(R)-346	Tyr(Y)-91	2.6
	Arg(R)-346	Gly(G)-104	2.6
	Lys(K)-444	Asp(D)-105	2.6
Salt bridge	Lys(K)-444	Asp(D)-105	2.6
Cation-□	Arg(R)-346	Trp(W)-32	3.8

Residues in light green cells are in light chain of BA7208 Fab, Residues in dark green cells are in heavy chain of BA7208 Fab.

Table S4. Protein-protein interaction between Delta RBD and BA7125 Fab.

	Residue in Delta RBD	Residue in BA7125 Fab	Distance (Å)
Hydrogen bond	Arg(R)-403	Asp(D)-94	3.3
	Lys(K)-417	Asp(D)-94	3.3
	Tyr(Y)-453	Gly(G)-93	3.4
	Lys(K)-458	Gly(G)-31	2.8
	Gly(G)-476	Tyr(Y)-32	2.6
	Tyr(Y)-489	His(H)-101	2.6
	Phe(F)-490	Tyr(Y)-33	3.3
	Tyr(Y)-505	Glu(E)-1	2.4
Salt bridge	Arg(R)-403	Asp(D)-94	3.3
	Lys(K)-417	Asp(D)-94	3.3

Residues in light yellow cells are in light chain of BA7125 Fab, Residues in dark yellow cells are in heavy chain of BA7125 Fab.

Table S5. Cryo-EM data collection, refinement and validation statistics for the structure of Delta S with BA7054-Fab and BA7125-Fab.

Delta S-BA7054/BA7125 Consensus map	
**Data collection and processing**	
Microscope	FEI Titan Krios
Camera	Gatan K3
Magnification	46656
Voltage (kV)	300
Electron exposure ($e^{-}/Å^2$)	50
Defocus range (μm)	-1.0 to -2.0
Pixel size (Å)	1.072
Initial particles images	1,659,975
Final particles images	335,175
Symmetry imposed	C1
**Map resolution (Å)**	3.21
FSC threshold	0.143
Map resolution range (Å)	2.0-6.5
**Refinement**	
Initial model used (PDB code)	6XCN
Map sharpening *B* factor ($Å^2$)	-120
**Model composition**	
Non-hydrogen atoms	39033
Protein residues	4998
Ligands	36
**R.m.s. deviations**	
Bond lengths (Å)	0.004
Bond angles (°)	0.826
**Validation**	
MolProbity score	3.03
Clashscore	9.88
Poor rotamers (%)	0.28
**Ramachandran plot**	
Favored (%)	94.42
Allowed (%)	5.58
Disallowed (%)	0.00

Table S6. Protein-protein interaction between Delta RBD and BA7054 Fab.

	Residue in Delta RBD	Residue in BA7054 Fab	Distance (Å)
Hydrogen bond	Arg(R)-346	Ala(A)-91	2.3
	Arg(R)-346	Thr(T)-103	3.1
	Asn(N)-440	Asp(D)-54	2.1
	Asn(N)-440	Asn(N)-57	3.1
	Lys(K)-444	Asp(D)-105	2.7
	Lys(K)-444	Asp(D)-106	3.1
Salt bridge	Lys(K)-444	Asp(D)-105	2.7
	Lys(K)-444	Asp(D)-106	3.6

Residues in light pink cells are in light chain of BA7054 Fab, Residues in dark pink cells are in heavy chain of BA7054 Fab.

Table S7. Cryo-EM data collection, refinement and validation statistics for the structure of Omicron S with BA7208-Fab.

	Omicron S-BA7208 Consensus map
**Data collection and processing**	
Microscope	FEI Titan Krios
Camera	Gatan K3
Magnification	81,000
Voltage (kV)	300
Electron exposure (e^{-}/Å^2)	50
Defocus range (μm)	-1.0~-2.0
Pixel size (Å)	0.856
Frames/movie	32
Movies (total)	2,554
Initial particle images (no.)	633,788
Final particle images (no.)	439,731
Symmetry imposed	C1
**Map resolution (Å)**	2.62
FSC threshold	0.143
Map resolution range (Å)	1.8-4.5
**Refinement**	
Initial model used (PDB code)	AF2 predicted
Model resolution (Å)	2.62
FSC threshold	0.143
Model resolution range (Å)	1.8-4.5
Map sharpening *B* factor (Å^2)	-97.2
**Model composition**	
Protein residues	3,296
**R.m.s. deviations**	
Bond lengths (Å)	0.003
Bond angles (°)	0.556
**Validation**	
Clashscore	9.23
Poor rotamers (%)	0
**Ramachandran plot**	
Favored (%)	98.18
Allowed (%)	1.82

Disallowed (%)	0

Table S8. Protein-protein interaction between Omicron BA.1 RBD and BA7208 Fab.

	Residue in Omicron RBD	Residue in BA7208 Fab	Distance (Å)
Hydrogen bond	Thr(T)-345	Phe(F)-94	3.2
	Arg(R)-346	Tyr(Y)-91	3.4
	Arg(R)-346	Asp(D)-92	2.8
	Arg(R)-346	Gly(G)-104	2.8
	Lys(K)-440	Trp(W)-34	2.9
	Lys(K)-440	Asp(D)-56	3.5
	Ser(S)-443	Gly(G)-103	3.7
	Lys(K)-444	Asp(D)-105	2.6
Salt bridge	Arg(R)-346	Asp(D)-92	3.6
	Lys(K)-440	Asp(D)-56	3.5
	Lys(K)-444	Asp(D)-105	2.6
Cation-□	Arg(R)-346	Trp(W)-32	3.6

Residues in light green cells are in light chain of BA7208 Fab, Residues in dark green cells are in heavy chain of BA7208 Fab.

Table S9. PK parameters of single intravenous administration of BA7208, BA7125 and BA7208/7125 in mice (n=3).

PK Parameters	BA7208		BA7125		BA7208/7125	
	Mean	SD	Mean	SD	Mean	SD
$T_{1/2}$(hour)	94.215	43.674	127.749	15.723	100.494	10.433
T_{max}(hour)	0.083	0.000	0.083	0.000	0.083	0.000
C_{max}(μg/mL)	215.748	25.166	184.160	22.316	194.473	12.921
C_0(μg/mL)	231.827	27.323	197.348	23.664	209.692	16.702
AUC_{0-t}(hour*μg/mL)	8577.179	3127.581	9990.807	1195.331	7293.982	1635.068
$AUC_{0-\infty}$(hour*μg/mL)	9695.235	4029.493	11778.903	1960.283	8468.373	1247.218
Vz(mL/kg)	137.960	22.400	157.326	7.891	172.773	23.143
Cl(mL/hour/kg)	1.205	0.633	0.867	0.159	1.197	0.164
MRT_{0-t}(hour)	142.535	59.431	180.680	17.756	127.411	12.514
